# At home e-based physical exercise programs in patients with multiple sclerosis: a scoping review

**DOI:** 10.3389/fneur.2024.1449519

**Published:** 2024-10-15

**Authors:** Rafl Adnan, Stine Gundtoft Roikjaer, Sara Samadzadeh, Camilla Josefine Richter, Brian G. Weinshenker, Friedemann Paul, Søren Thorgaard Skou, Ulrik Dalgas, Nasrin Asgari

**Affiliations:** ^1^Institute of Regional Health Research, University of Southern Denmark, Odense, Denmark; ^2^Institute of Molecular Medicine, University of Southern Denmark, Odense, Denmark; ^3^The Center for Neurological Research, Department of Neurology, Slagelse Hospital, Slagelse, Denmark; ^4^The Research and Implementation Unit PROgrez, Department of Physiotherapy and Occupational Therapy, Næstved-Slagelse-Ringsted Hospitals, Slagelse, Denmark; ^5^Charité – Universitätsmedizin Berlin, Corporate Member of Freie Universität Berlin and Humboldt-Universität zu Berlin, Experimental and Clinical Research Center, Berlin, Germany; ^6^Department of Neurology, University of Virginia, Charlottesville, VA, United States; ^7^Experimental and Clinical Research Center, A Cooperation Between the Max Delbrück Center for Molecular Medicine in the Helmholtz Association and Charité Universitätsmedizin Berlin, Berlin, Germany; ^8^Max Delbrück Center for Molecular Medicine in the Helmholtz Association (MDC), Berlin, Germany; ^9^NeuroCure Clinical Research Center, Charité – Universitätsmedizin Berlin, Corporate Member of Freie Universität Berlin and Humboldt-Universität zu Berlin, Berlin, Germany; ^10^Center for Muscle and Joint Health, Department of Sports Science and Clinical Biomechanics, University of Southern Denmark, Odense, Denmark; ^11^Exercise Biology, Department of Public Health, Aarhus University, Aarhus, Denmark

**Keywords:** multiple sclerosis, physical exercise, e-based rehabilitation at home, e-based physical exercise, rehabilitation

## Abstract

**Introduction:**

Physical exercise (PE) improves symptoms and quality of life in people with multiple sclerosis (pwMS). However, incorporating PE into daily lives of pwMS pose difficulties. As an alternative to in-person PE, e-based PE has been proposed because of its advantages in terms of accessibility and convenience.

**Objective:**

To synthesize existing evidence on e-based PE at home in adults MS and discuss study designs, rehabilitation programs, intervention effects and possible knowledge gaps.

**Methods:**

In this scoping review, a systematic search in electronic databases including Embase, Medline, CINAHL and Cochrane Library was conducted following the PRISMA guidelines. Peer-reviewed articles in English on e-based PE interventional studies at home involving adult pwMS, published from 2008 until April 2023, were identified and exported to Covidence. Data from the included studies were extracted and synthesized. This scoping review identified different types of e-based PE interventions used in pwMS across different study designs, but when evaluating intervention effects, this review focused solely on randomized controlled trials (RCTs).

**Results:**

A total of 3,006 studies were retrieved and 179 studies were screened in full text, resulting in the inclusion of 54 studies with a total of 2,359 pwMS. Of those, 33 were RCTs and three were qualitative studies. The studies reported on various e-based interventions including video-based programs, telerehabilitation, and web-based programs. The interventions evaluated muscle strength, cardiorespiratory fitness, walking speed, endurance, balance, and fatigue, as well as symptoms of depression and cognitive dysfunction. E-based PE interventions at home in RCTs demonstrated improvement of depressive symptoms and anxiety, while inconsistent results were reported for fatigue, walking speed and balance. No significant benefits were observed regarding dexterity. Results were generally heterogeneous and were limited by small sample sizes. Several limitations were identified, such as lack of physical activity assessment prior to the intervention and poor reporting of duration, intensity, frequency and adherence to e-based PE interventions.

**Conclusion:**

E-based PE interventions in pwMS may improve MS-related symptoms, but the study quality is generally low, and findings are often inconsistent. Several important limitations of the existing literature have been identified in the present review, which can guide future research.

## 1 Introduction

Multiple sclerosis (MS) is an inflammatory, demyelinating disease of the central nervous system (CNS), considered to be immune-mediated (1). MS is characterized by chronic inflammation and destruction of the myelin sheath of axons, which may lead to neuroaxonal degeneration (2), resulting in clinical and irreversible neurological disability (3–5).

Current management of MS is non-curative, and approved MS immunotherapies, also called disease-modifying therapies (DMT), act on the inflammatory component of the disease and efficiently reduce clinical and radiological relapses in relapsing-remitting MS (RRMS) (6). However, these therapies do not sufficiently prevent disease progression and disability accrual. RRMS often progresses to secondary progressive MS (SPMS), characterized by gradual worsening of neurologic function, and at this point only limited therapeutic options beyond symptomatic treatments and physiotherapy exist (4).

Evidence has emerged that physical exercise (PE) can serve as symptomatic treatment for several chronic diseases, including MS (7–10). In people with MS (pwMS), PE directly improves muscle strength and cardiorespiratory fitness (11–13) as well as walking speed, endurance (14–16), and balance (17, 18). A reduction in fatigue (19–27), symptoms of depression (22, 27–30) and improvements in cognitive dysfunction (31) and quality of life after PE have also been reported (32–34). Furthermore, PE reduces the rate of relapses in RRMS and may therefore have a disease-modifying effect (35). This may relate to PE being able to positively alter inflammation, as evidenced by improvement in levels of peripheral inflammatory markers, such as TNF-alfa, INF-gamma, IL-4, and IL-10 (36–38).

Despite the apparent benefits for pwMS, a notable number of participants in PE programs either do not initiate, discontinue or struggle to sustain PE (39, 40). This highlights the challenge of integrating and sustaining physical activity (PA) in the daily lives of people with a chronic condition like MS. The primary purpose of PE therapy is to increase the level of PA in pwMS and represents a major effort in prevention and rehabilitation of pwMS. Some of the barriers to pwMS for sustaining PE intervention may include lack of time, long distances to health centers and a higher degree of disability (41, 42), and there is a need to identify more convenient and accessible interventions or delivery models. One such alternative delivery model is home-based electronic health (eHealth) intervention/e-based PE (43–45), which can be defined as, “PE delivered via a digital health intervention such as a computer, mobile or a similar media device; through platforms such as internet websites or web applications at home/residence to support the achievement of health objectives” (46). Literature reviews focusing on telerehabilitation programs specifically tailored to pwMS are few and often focus on distinct outcome measurements (47), or are focused only on rehabilitation of the upper limbs or gait and balance (48, 49). A broader analysis of experimental evidence on digital, home-based PE in pwMS can provide an overview on the effectiveness of rehabilitation programs on physical and mental symptoms in pwMS. This information may identify knowledge gaps and provide care recommendations. The aim of this scoping review was to summarize comprehensively and systematically the current evidence on the different types of e-based PE at home and their effects in pwMS, subsequently discuss possible knowledge gaps, which may be found in study designs, feasibility, assessment of outcomes and adherence to intervention.

## 2 Materials and methods

This review followed the Preferred Reporting Items for Systematic Reviews and Meta Analysis extension for Scoping Reviews (PRISMA-ScR) Checklist (50). Screening and data extraction were performed using Covidence (https://www.covidence.org) and Microsoft Excel, respectively. A comprehensive literature search was conducted to identify published literature evaluating at home e-based PE in pwMS, with no date restrictions for article retrieval. Home-based eHealth interventions were defined as PE delivered via a digital health intervention performed at home/residence.

### 2.1 Eligibility criteria

Table 1 presents a structured presentation of the inclusion and exclusion criteria applied in this scoping review of e-based PE intervention studies in adult (18+) pwMS. At home e-based PE interventional studies including randomized controlled trials (RCTs) and other clinical research designs as well as qualitative studies, written in English, and with available full-texts were included. Studies that did not use e-based PE at home were excluded. Editorials, opinion pieces, magazine/newspaper articles, case reports, reviews, protocols, survey studies, poster abstracts, and conference abstracts were also excluded.

**Table 1:** Study selection criteria.

**Criterion**	**Inclusion criteria**	**Exclusion criteria**
Patient population	Diagnosis of MS	–
	Age ≥18 years	
	No sex or gender restrictions	
Intervention	At home e-based physical exercise interventional studies as well as qualitative studies	Editorials, opinion pieces, magazine/newspaper articles, case reports, reviews, protocols, survey studies, poster abstracts, and conference abstracts not published in full
	No publication restriction	
	Comparator	Usual care, non-digital interventional controls, other digital interventional controls, healthy controls	
Outcome	No restrictions in outcomes	
Time	No duration restriction on interventions and follow-up	–
Setting	No geographical restrictions	

### 2.2 Search strategy and information sources

The Literature search was conducted, with no time restriction, in the electronic databases Embase, Medline, CINAHL and Cochrane published from 2008 until April 2023. Keywords included “multiple sclerosis,” “exercise,” and “telerehabilitation.” Details of the search are available in Supplementary Document 1. The search strategy was discussed within the research group and was evaluated by a research librarian. Subsequently, relevant articles including reference lists, and published reviews were selected based on eligibility criteria as defined in Table 1.

### 2.3 Selection of sources of evidence

Articles from the databases were exported to Covidence, which was used in the screening process. Articles were screened for titles, abstracts as well as full texts.

### 2.4 Data charting process

Title- and abstract-screening, along with full-text reviews, were conducted by two independent reviewers: screening by title and abstract as well as full-text was done by RA for all studies, and a second screening was performed by either CJR, SS or PB. Conflicts were deliberated, and consensus was sought. In cases where agreement could not be achieved, a third reviewer was engaged (SGR). Data extraction was similarly performed independently by RA, and a second extraction by either CJR or SS. Upon encountering disagreements, the pertinent studies were re-examined to resolve any conflicts.

A tailored extraction sheet was made in Microsoft Excel, and it was tested by two reviewers on 5 articles. Extractions were compared, and necessary modifications were applied to the extraction template.

### 2.5 Data items

We extracted data on demography, clinical phenotypes and characteristics of adult pwMS as well as study designs, sample size, intervention characteristics, length of follow-up, study parameters (MS-related outcomes and assessment), intervention characteristics, effects of the intervention and data on adherence and drop-out. The data extraction process was double-checked to ensure the accuracy of data collection.

### 2.6 Synthesis of results

The studies were grouped by the type of digital platform used (i.e. web-based, apps etc.). Hereafter, we summarized the number of different study designs, type of physical exercise and type of control group used. Then the applied outcome measures were grouped by outcome. Finally, the outcomes of all the RCTs were synthesized.

## 3 Results

**Figure 1:**
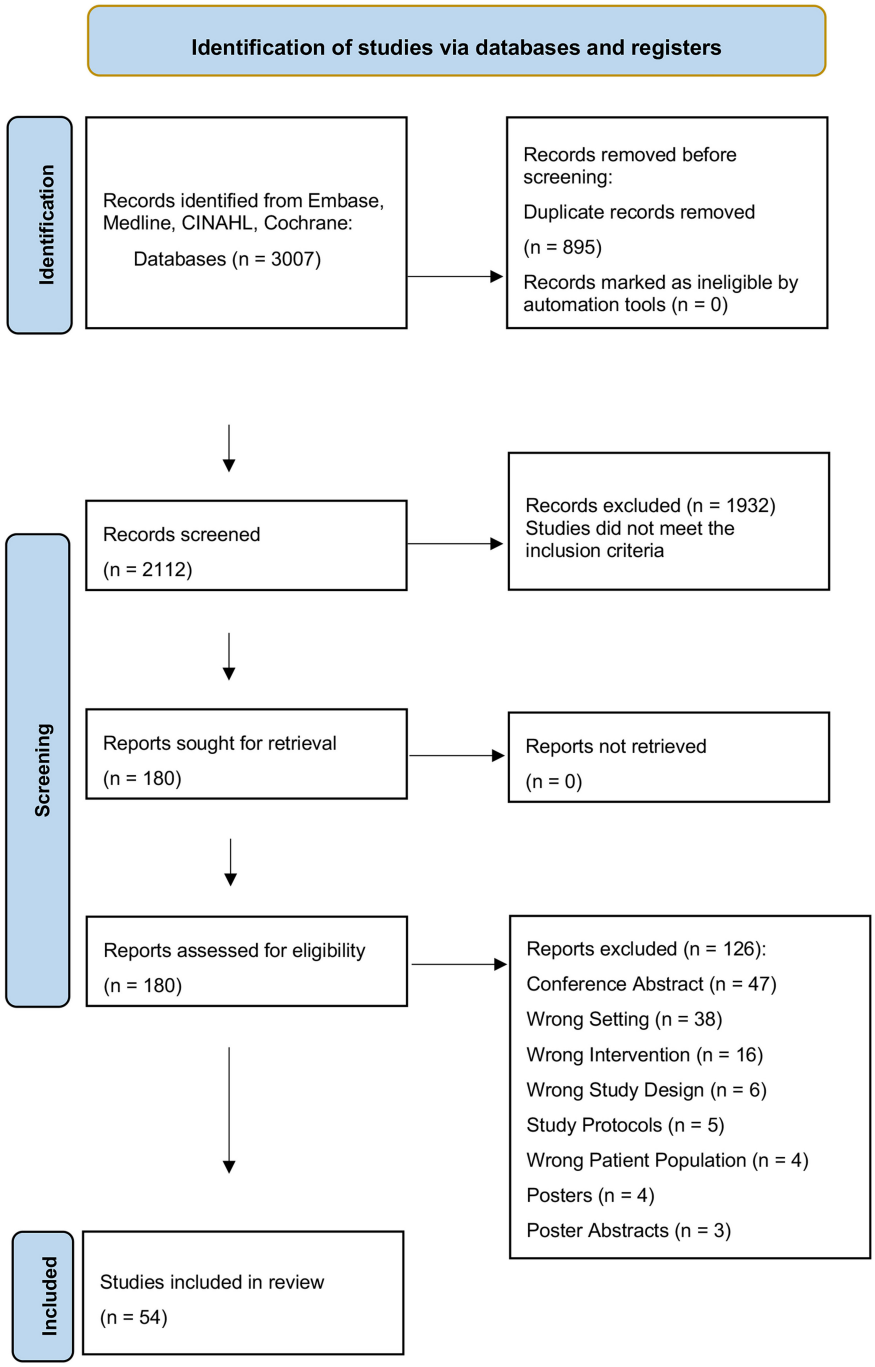
Flowchart of study selection process following the PRISMA flowchart: flowchart depicting the study selection process for a systematic review, showing initial screening, exclusion of duplicates, relevance screening, full-text assessment, and the final inclusion of 54 studies.

Figure 1 depicts the PRISMA flow diagram which presents the sequence of information through the various phases of the review. From our database searches of the mentioned databases, a total of 3,006 studies were retrieved. 895 duplicates were removed, and thus, 2,111 studies were screened based on their titles and abstracts. Of these, 1,922 were excluded for not providing information on MS and/or e-based PE. The remaining 179 studies were assessed in full text, resulting in the selection of 54 studies for inclusion with a total of 2,359 pwMS. Exclusions were primarily due to articles being conference abstracts, and for studies evaluating e-based PE interventions performed away from home. The included studies were all published between 2008 and 2023.

### 3.1 Characteristics of studies

#### 3.1.1 Study designs

**Figure 2:**
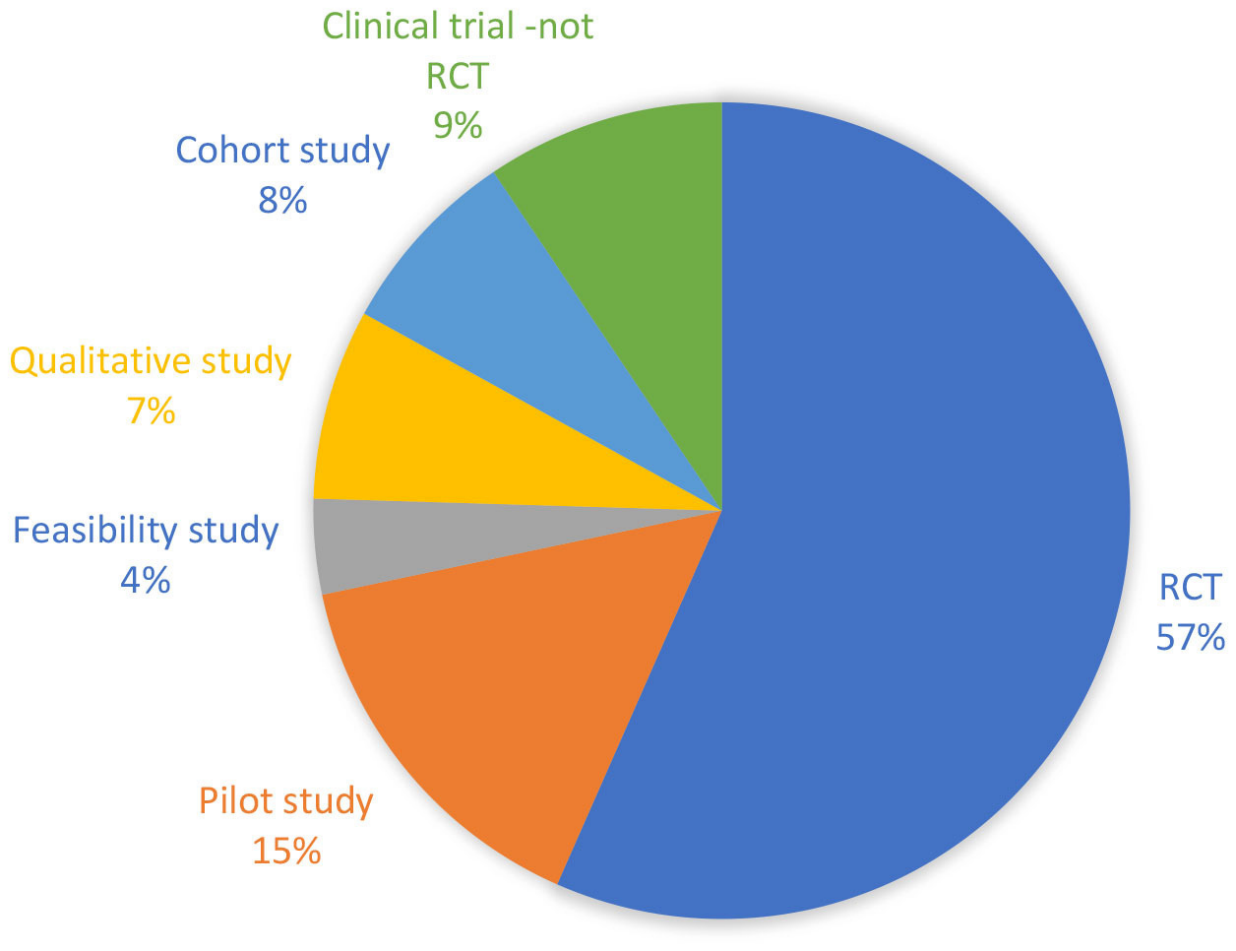
Pie diagram showing the relative distribution of study designs of the included studies.

Among the 54 studies, 33 were randomized controlled trials (RCTs), and three were qualitative studies. The qualitative studies were interview studies. The study by Knox et al. (51) used a reflexive thematic analysis, the study by Dennett et al. (52) used thematic analysis, while the study by Plow et al. (53) used an inductive-category and theme-development approach to analyse data. For detailed characteristics of the included studies (see Appendix 1). The diverse designs of the remaining studies are detailed in Figure 2.

**Figure 3:**
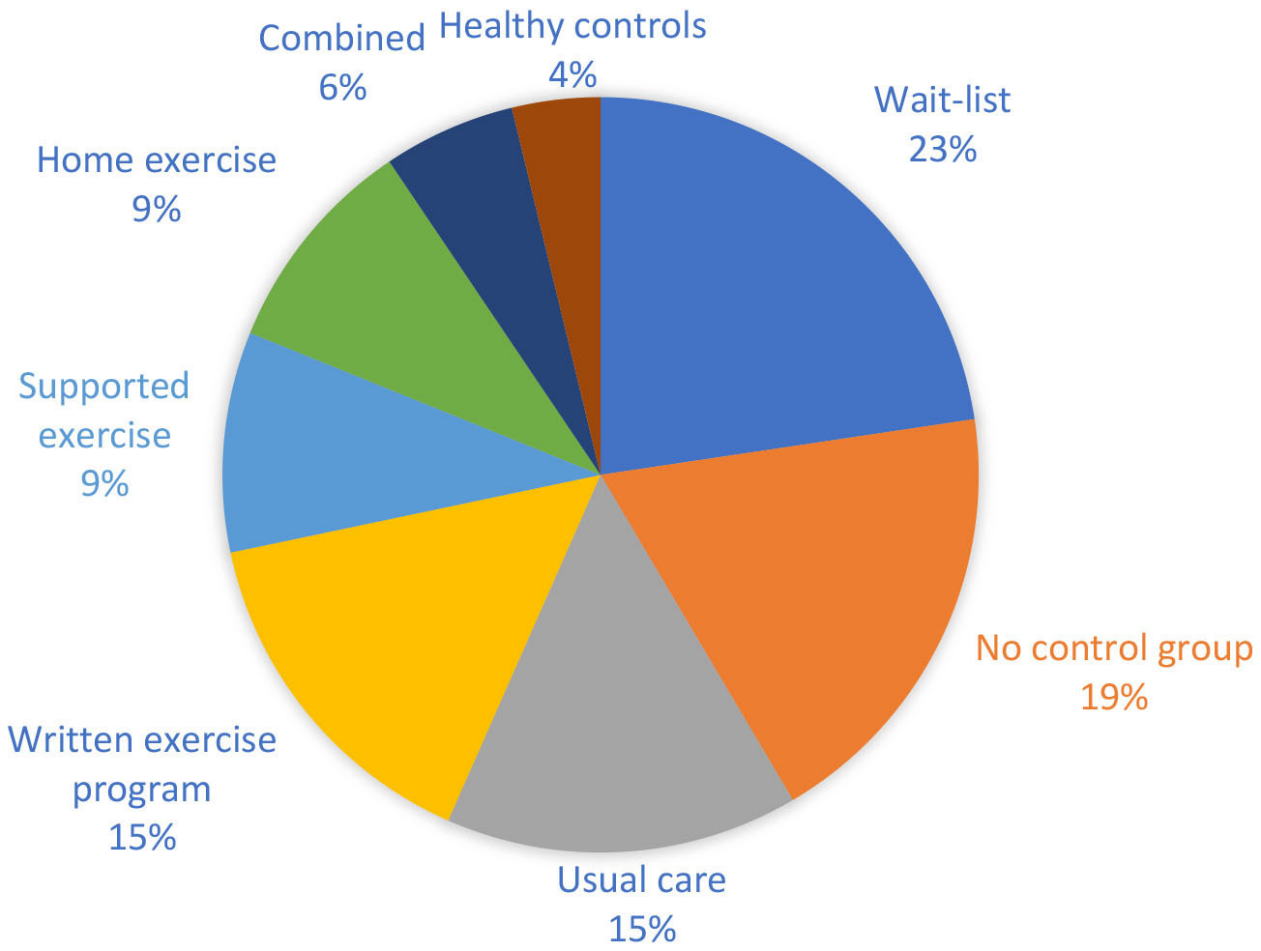
Pie diagram showing the relative distribution of control groups in the included studies.

A total of 42 of the included studies had a control group. Of those 42 studies 33 were RCTs and 9 were non-randomized, controlled trials. Seven studies included MS controls who received usual care (54–58), 12 included controls that received home-based exercise programs that were not delivered e-based (51, 52, 59–68), and 12 of the studies included waiting-list controls (19, 69–78), while four of the studies included control groups that received supervised PE (Figure 3) (79–82). A total of 49 studies included pwMS as controls except three that employed healthy controls (42, 83, 84). Four of the studies included more than one type of control group (83–86).

The diverse designs and uneven control groups complicated synthesis of the findings, evaluation of intervention efficacy and the global application to MS populations.

#### 3.1.2 Study populations

All studies provided information on sex and age except for the study by Hermens et al. (58), which did not report on sex, and the study by Knox et al. (51), which failed to specify their distribution by sex. Four studies only included female pwMS (87), while the remainder included both male and female subjects. In total, 986 females and 284 males were enrolled into the intervention groups across the included studies.

In the experimental groups the mean age ranged from 35.3 (±8.6) to 63.8 (±4.1) years (66, 77), while the control groups had a mean age between 34.8 (±5.6) and 65.1 (±5.2) years (66, 84). Additionally, one study reported an age range of 28–68 years in the experimental group (52). Among the studies that provided information of median age, the experimental group's median age ranged from 34.5 to 57.5 years (51, 83), while one study in the control group reported a median age of 31 years (83).

Variation in reporting of sex and age across studies challenges the generalizability of outcomes, and inconsistencies in data complicate group comparisons, as will be discussed.

#### 3.1.3 MS phenotypes

Forty-four of the included studies reported on the MS phenotype. Thirteen of these studies included person with RRMS as well as primary progressive MS (PPMS) and SPMS (19, 51, 52, 60, 65, 73, 79, 81, 82, 88–91), while seven studies exclusively focused on RRMS and SPMS (63, 67, 69, 74, 77, 84, 92). One study included RRMS as well as progressive pwMS without specifying whether it was primary or secondary MS (59). Two of the studies did not include persons with RRMS, one included persons with PPMS and SPMS (93), the other included persons with progressive MS without specifying the phenotype further (61).

#### 3.1.4 Time since diagnosis of MS

In all studies, the time since diagnosis was reported. The mean time since diagnosis ranged from 6.3 to 21.9 (±10.7) years in the experimental group (66, 71), and from 6.2 (±3.96) to 20.1 (±13.0) years in the control group (61, 94). Regarding the studies that reported on the median, there was a range in median of four to 19 years in the experimental group (51, 82). Thomas et al. (95) divided the time since diagnosis into different categories.

The reported data suggests that the studies encompassed a wide spectrum of MS progression stages, which could influence the outcomes of physical exercise interventions.

#### 3.1.5 Disability status

Thirty-five of the included studies used the Expanded Disability Status Scale (EDSS) and nine used the Patient Determined Disease Steps (PDDS) to report disability status. In 12 studies, neither EDSS nor PDDS were reported. The mean EDSS ranged from 1.9 (±1.1) to 6 (±0.5) in the experimental group (42, 55, 56), and from 2.6 (±1.0) to 5.8 (±0.5) in the control group (55, 56, 87). The median EDSS ranged from 1 to 6 in the experimental group (64, 83), and from 2 to 6 in the control group (64, 83). For PDDS, the mean ranged from 3.7 (±1.1) to 4.5 (±1.6) in the experimental group (89, 96), and 3.9 (±1.4) to 4.8 (±1.7) in the control group (62, 89), while the median PDSS ranged from 1 to 4 in the experimental group (51, 72), and from 1 to 3 in the control group (72, 73). The remaining studies reported on EDSS and PDSS as a range of reporting them in category format.

The means and medians of EDSS and PDDS scores were closely aligned between experimental and control groups, indicating a homogeneous disability severity among participants. This uniformity allows more direct comparisons of intervention outcomes across studies.

### 3.2 The study interventions

#### 3.2.1 Type of digital delivery of the PE intervention

Appendix 2 presents detailed data on the interventions of the included studies.

**Figure 4:**
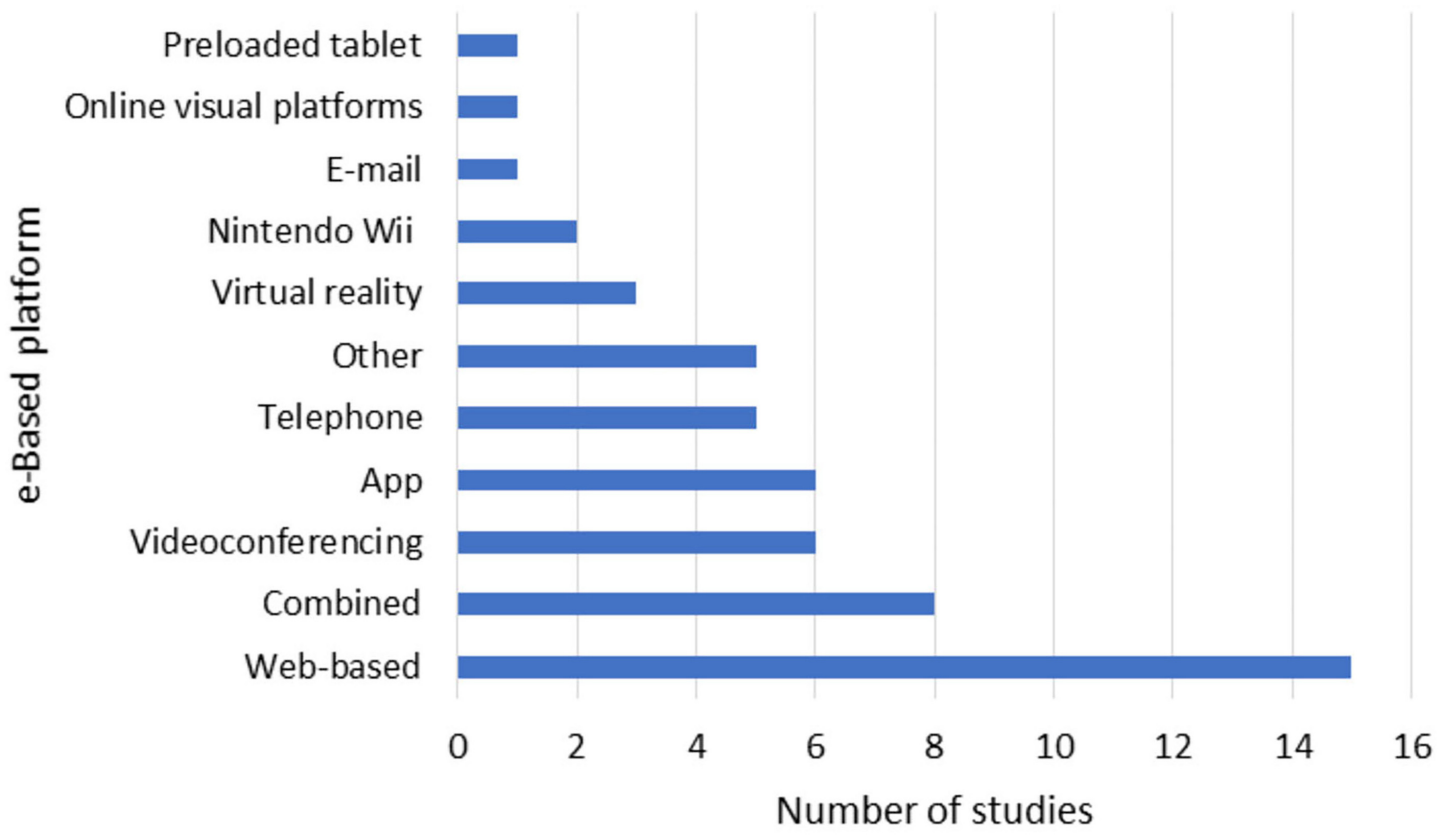
Distribution of the type of digital platform used in the included studies.

A total of 15 studies delivered the e-based at home PE intervention via a website, seven through an app, five via telephone, another five via videoconferencing, three used virtual reality, and one used email. Eight studies used other platforms for delivering the PE intervention (see Figure 4).

In the study by Huijgen et al. (76), the intervention was delivered via the Home Care Activity Desk, a portable device monitoring at home PE. Prosperini et al. (77, 84) used a Nintendo Wii Balance Board System. Thomas et al. (95) and Plow et al. (53, 97) utilized Nintendo Wii. Kim et al. (98) employed tablets. Hermens et al. (58) used a home care activity desk, and Jeong et al. (99) used telerehabilitation without further specifying the platform.

In total, 11 studies combined different platforms for intervention delivery. Plow et al. (75, 85) used videoconferencing as well as telephone. Fleming et al. (86) used DVD and telephone. Manns et al. (91) utilized videoconferencing or telephone along with newsletters. Najafi et al. (87) employed online visual platforms including Google meet, Zoom and Instagram. Pilutti et al. (35) combined a website, videoconferencing, and an electronic spreadsheet for step count tracking.

Learmonth et al. (74) employed DVD material, calendars, logbooks, videoconferencing and newsletters. Dlugonski et al. (72) utilized a website and videoconferencing. Mardaniyan Ghahfarrokhi et al. (68) used email, WhatsApp, SMS, phone calls, WhatsApp video calls, and DVDs. Sandroff et al. (100) used a website, a goal tracker software as well as videoconferencing.

In summary, studies on e-based PE interventions employed a diverse set of technologies, including websites, apps, telephones, videoconferencing, and virtual reality, to facilitate home-based participant engagement. The integration of multiple digital platforms aimed to enhance interaction, monitoring, and feedback, potentially improving program adherence and effectiveness. Due to heterogeneity of reporting it was not clear if the choice of platform influenced compliance/adherence.

#### 3.2.2 Exercise interventions

A total of 44 of the studies reported on the type of PE administered to the subjects (15, 19, 42, 51, 52, 54–56, 58–64, 67–69, 71, 73, 75–77, 79–94, 96, 98, 99). Ten studies provided personalized PE programs (52, 54, 56, 62, 64, 89, 94, 96, 99). Three studies focused on dexterity training (60, 88, 90). Pilates was the form of PE in two studies (67, 86), while another two studies incorporated both yoga and pilates (87, 98), with one study also including neurorehabilitation activities such as dual-tasking and functional tasks (98). Walking was the chosen PE intervention in four studies (19, 35, 75, 92), while one study combined walking with cycling and lower extremity strength training (80). For details on the other studies (see Appendix 2). All studies report on the duration of the intervention, ranging from four weeks (88) to 12 months (95). All but 13 studies did not specify the frequency of the PE sessions.

The reviewed studies demonstrate a variety in PE interventions for subjects, from personalized programs to specific exercises, like pilates, yoga, and walking, indicating tailored approaches to fit diverse needs. The interventions lasted from four weeks to 12 months, yet many studies lacked details on session frequency, highlighting a gap in reporting that could impact the assessment and interpretation of PE effectiveness.

### 3.3 Outcome measurements and findings

#### 3.3.1 Assessments

Most of the studies included in this review used an online website and electronic questionnaire to assess e-based PE at home; in three cases performed in participants' homes (63, 89, 97), one combined with a visit at the clinic (96), nine were performed at the clinic, with online platform and with a questionnaire at the clinic (58, 64, 66, 71, 76, 79, 82, 91, 95). Assessment of e-based PE at home were performed by clinicians and healthcare professionals in combination with reports from participants.

#### 3.3.2 Measurement of physical activity

Various methods exist for measuring frequency and intensity of PE performed by subjects outside the PE offered via the intervention. Of the 54 included studies, only 14 used a tool to measure PE beyond the exercise delivered through the intervention. Four studies employed the Godin Leisure-Time Exercise Questionnaire (GLTEQ) to determine the frequency and intensity of exercise during free time in a typical week (61, 72, 86, 101). Two studies used accelerometers to measure physical activity (PA) (64, 91), while five studies used both GLTEQ and accelerometers (19, 35, 75, 85, 95). Three studies adopted the International Physical Activity Questionnaire (IPAQ) for this purpose (87, 92, 100).

The measurement of PA outside of intervention-provided exercises in e-based MS exercise studies varied, with only a fraction of studies employing tools like the GLTEQ, accelerometers, or the IPAQ.

#### 3.3.3 Assessments of walking capacity

Twenty-three studies included walking assessments as an outcome measure. Eight studies used the Timed 25-Foot Walk Test (T25FW) (55, 56, 62, 64, 65, 87), two used the Twelve Item MS Walking Scale (MSWS-12) (57, 72), and various combinations of the Two Minute Walk Test (2MWT), Six Minute Walk Test (6MWT), Ten Meter Walk Test (10MWT), MSWS-12 and T25FW were used in the rest.

Conroy et al. (89) reported no significant changes using the T25FW between intervention and the control group. Kahraman et al. (83), employing the T25FW and MSWS-12, observed significant improvements in the intervention group after the 8 week intervention, while no significant changes were observed in the wait-list control group. Najafi et al. (87) found improvements in the T25FW, with tele-Pilates and tele-yoga groups showing enhanced walking speed post-intervention compared to the control group. Flachenecker et al. (54), using the 10mWT and 2minWT, reported significant improvement in both the intervention and the no-intervention control group, except for the 10mWT in the intervention group. Pagliari et al. (57), and Dlugonski et al. (72), both using the MSWS-12, found no significant improvements over time or between the telerehabilitation group and the usual care group. Most studies assessing walking outcomes in MS interventions reported significant improvements in walking capacity and speed, especially in intervention groups vs. controls (57, 72).

#### 3.3.4 Walking capacity and balance

Ten of the included studies measured balance, one measured gait, and three measured both balance and gait (Table 2).

**Table 2:** The different measurement instruments used for quantifying balance and/or gait.

**Assessment**	**Instrument**	**References**
Balance	The Berg Balance Scale (BBS)	(55, 56, 64, 65, 89)
Balance	The Step Test	(95)
Balance	The Activities-Specific Balance Confidence (ABC) Scale	(83)
Balance	The Timed Tandem Walk Test	(61)
Balance	The Tandem Stance Test & The Timed Tandem test	(68)
Gait	The Functional gait assessment (FGA)	(65)
Gait and balance	The Tinetti Score	(54)
Gait and balance	The Tinetti Score and the BBS	(82)
Gait and balance	The Dynamic Gait Index (DGI)	(83)

Abasiyanik et al. (67), using the Activities-Specific Balance Confidence (ABC) scale, found that balance was significantly improved in both the pilates and home exercise group post-intervention, but no significant changes between the groups were found. Conroy et al. (89), using the Berg Balance Scale (BBS) observed a significant worsening in balance with a home automated tele-management system vs. routine home rehabilitation. Flachenecker et al. (54), employing the Tinetti score, reported improved balance in both intervention and control groups post-intervention. Gutierrez et al. (82) noted significant improvement in the BBS in both the telerehabilitation group using video game-based virtual reality and the conventional rehabilitation program, control group, and significant improvement in the Tinetti score was only observed in the telerehabilitation group. A significant improvement in both the BBS and the Tinetti score was noted in the telerehabilitation group (82). Kahraman et al. (83), found significant improvement in the Dynamic Gait Index (DGI) in the telerehabilitation-based motor imaging training group post-intervention.

Interventions on balance show mixed results, with improvements in some cases and no change or worsening in others, highlighting the complexity of their impact.

#### 3.3.5 Ataxia

Only one study assessed ataxia, using the rater-assessed “kinetic functions sub-parameter” of the International Cooperative Ataxia Rating Scale (K-ICARS) (81). Dogan et al. (81) found that both the virtual reality supported task-oriented circuit therapy groups (V-TOCT) and the mobile application-based telerehabilitation (TR) group exhibited significant improvements in the K-ICARS post-intervention, but between group significant improvement was also observed in the V-TOCT group. It was concluded that digital rehabilitation methods like virtual reality and mobile apps show promise in improving ataxia (81).

#### 3.3.6 Dexterity

In total, 11 of the included studies measured dexterity. Nine studies used the Nine-Hole Peg (9HPT) test to measure dexterity (57, 58, 60, 61, 76, 88, 90, 92, 95), one used the Box and Block (BBT) test (57), and another used the Minnesota Manual Dexterity Test (MMDT) (81)

Pagliari et al. (57), using the BBT, reported significant improvements in both the home-based virtual reality rehabilitation system training group and the usual care group. Van Beek et al. (60), employing the 9HPT, found no significant difference between the tablet app-based dexterity training group (TAD-MS) and a theraband group. In the study by Hermens et al. (58), using the 9HPT, the differences between intervention and control groups remained unclear due to the widths of the confidence intervals being outside the equality bounds.

The studies on dexterity present mixed results; interventions like virtual reality show potential for improvement, whereas others, like tablet app-based training, reveal no significant changes. This underscores the complexity of improving dexterity in pwMS via digital methods.

#### 3.3.7 Fatigue and pain

In total, 16 of the included studies measured fatigue, and one study measured pain. Table 3 shows the different measurements instruments used for fatigue.

**Table 3:** The different measurement instruments used for quantifying fatigue.

**Instrument**	**References**
The Modified Fatigue Impact Scale (MFIS)	(63, 65, 86, 93, 97, 101)
The Würzburg Fatigue Inventory for Multiple Sclerosis (WEIMuS)	(54, 69)
The Fatigue Severity Scale (FSS),	(57, 94)
The Fatigue Impact Scale (FIS)	(75, 85)
The Fatigue Symptom Inventory (FSI)	(95)
The MFIS and FSS	(19, 92)

Turner et al. (101), using the Modified Fatigue Impact Scale (MFIS), observed fatigue improvement in the telephone counseling group compared to a self-directed education group. Plow et al., utilizing the Fatigue Impact Scale (FIS), found that PA delivered through group teleconferences and tailored phone calls, combined with fatigue management, improved fatigue compared to the contact-control (75).

Plow et al. (75), using the FIS, reported significant improvements in the PA plus fatigue self-management group compared to the contact-control group post intervention, with no significant differences at 26 weeks post-randomization follow-up. Pilutti et al. (19), employing both MFIS and the Fatigue Severity Scale (FSS), demonstrated decreased fatigue in the internet-delivered behavioral intervention group, noting no significant pain differences using Short-Form McGill Pain Questionnaire (SF-MPQ).

Tarakci et al. (94), using FSS, and Flachenecker et al. (54) using the Würzburg Fatigue Inventory for Multiple Sclerosis (WEIMus), both reported improvements in fatigue with varied long-term effects post-intervention.

These studies show diverse impacts on fatigue, with notable improvements in some cases with telephone/online counseling.

#### 3.3.8 Cognition

Eight of the included studies assessed cognition. Table 4 shows the different instruments used to measure cognition Table 5.

**Table 4:** The different measurement instruments used for quantifying QoL in home e-based studies on PE in pwMS.

**Measurement instrument used**	**References**
The 36-items Short Form Survey (SF-36)	(58, 65, 92, 97)
The Multiple Sclerosis Quality of Life-54 (MS QoL-54) Scale	(57, 87, 96, 99)
The Leeds Multiple Sclerosis Quality of Life Scale	(55, 56)
The Hamburg Quality of Life Questionnaire in Multiple Sclerosis (HAQUAMS)	(61, 69)
The Five-Level EuroQoL-5 dimensions health state utility scale (EQ-5D-5L)	(93)
The Quality-of-Life Scale (QoLS)	
SF-36 and EQ-5D-5L	(95)

**Table 5:** The measurement instruments used for quantifying cognition.

**Instrument**	**References**
The Symbol Digit Modalities Test (SDMT)	(61, 66, 68, 92, 100)
The Brief International Cognitive Assessment for Multiple Sclerosis (BICAMS)	(67)
The Montreal Cognitive Assessment (MoCA) and the SDMT	(57)

The study by Sandroff et al. (100), which used the Symbol Digits Modalities Test (SDMT), observed increased scores in the intervention group. The study by Pagliari et al. (57), which used both the SDMT and the Montreal Cognitive Assessment (MOCA), noted a comparable effect on cognition in both the intervention and the control groups.

The study by Sebastiao et al. (66), which used SDMT, found no significant effect on cognition in the home-based Square-Stepping Exercise group. The study by Abasiyanik et al. (67), which uses SDMT, reported that pilates-training programs improve cognition in comparison to the home-exercise, control group.

Cognitive assessment studies in interventions show mixed outcomes, with some indicating improvements and others reporting no significant changes compared to control groups, highlighting variability in cognitive response to interventions.

#### 3.3.9 Anxiety and depressive symptoms

Four of the included studies only measured depressive symptoms, while 10 studies assessed both depressive symptoms and anxiety. The Table 6 shows the different instruments used to measure anxiety and/or depressive symptoms.

**Table 6:** The different measurement instruments used for quantifying depression and/or anxiety.

**Measurement**	**Instrument**	**References**
Depression and anxiety	The Hospital Anxiety and Depression Scale (HADS)	(19, 55, 56, 59, 62, 64, 95)
Depression and anxiety	The Beck Depression Inventory (BDI) and the State-Trait Anxiety Inventory (STAI)	(57)
Depression and anxiety	the Hospital Anxiety and Depression Scale (HADS) and the STAI	(86)
Depression	The Hamilton Depression Scale (HAM-D)	(70, 71)
Depression	The Patient Health Questionnaire-9 (PHQ-9)	(101)
Depression	The BDI	(87)

Kratz et al. (70) and Bombardier et al. (71), utilizing the Hamilton Depression (HAM-D) Scale, noted significant improvement in depressive symptoms in the intervention group receiving telephone-based motivational interviewing in comparison to a waiting-list control group. Turner et al. (101), employing the Patient Health Questionnaire (PHQ-9), found improvements in the telephone counseling group compared to the education condition group. Pilutti et al. (19), using the Hospital and Anxiety Depression Scale (HADS), reported significant reduction in depression and anxiety symptoms in the web-based PA intervention group vs. the wait-list control.

Pagliari et al. (57), using the Beck Depression Inventory (BDI) and the State-Trait Anxiety Inventory (STAI), noted improvements in depressive symptoms in the home-based virtual reality rehabilitation as well as the usual care groups, with no significant difference between groups. Najafi et al. (87), using the BDI, found improvements in depressive symptoms in tele-pilates and tele-yoga groups, with no significant differences in the no-intervention control group.

The studies show that e-based interventions, including motivational interviewing and virtual reality, may improve depressive symptoms and anxiety in participants.

#### 3.3.10 Quality of life

Sixteen of the included studies measured health related quality of life (HRQoL). See table below for the various instruments used to measure QoL. Most studies only used one instrument to measure QoL while one study used two different instruments.

Pagliari et al. (57), employing the MS QoL-54 scale, observed significant improvement in the physical health composite of QoL with home-based virtual reality rehabilitation system training compared to a usual care group. Najafi et al. (87) used the MS QoL-54 scale, reported improved QoL in both the tele-yoga and tele-pilates groups vs. a no-intervention control group.

In the RCTs study by Jeong et al. (99), also using the MS QoL-54 scale, the authors found that physical telerehabilitation led to notable QoL improvements in the intervention group compared to a usual care control group. Tallner et al. (69), using the Hamburg Quality of Life Qestionnaire in MS (HAQUAMS), observed no QoL improvements in the web-based PE intervention group compared to the waiting-list control group. Tarakci et al., employing the Quality of Life Sclae (QoLS), found no differences in QoL between the structured supervised exercise group and the telerehabilitation group (94).

HRQoL outcomes in MS subjects participating in PE interventions vary, with some studies reporting QoL improvements while others report neutral findings.

#### 3.3.11 Participant reported outcome measures

In total, 15 studies used the MS impact scale 29 (MSIS-29) to evaluate physical and psychological impact of MS from the participants perspective (19, 54–56, 60, 62, 64, 72, 75, 77, 85, 90, 92, 95). Two studies reported on self-efficacy using the MS self-efficacy Scale (MSSE) to measure confidence in function and symptom management (65, 95). One study reported on self-efficacy using both the Spinal Cord Injury Exercise Self-Efficacy Scale (SCI-ESES) to assess confidence in performing physical activities and exercises as well as the MSSE (95).

One study used the University of Washington 6-item short form self-efficacy scale (UW-SES-SF) to assess confidence in managing MS (93). One study reported on self-efficacy using the Regulatory Emotional Self-Efficacy (RESE) Scale to assess confidence in regulating emotional states (57).

These studies utilize various scales, including MSIS-29, MSSE, ESES, UW-SES-SF, and RESE, to assess the disease's physical, psychological, and self-efficacy impacts.

#### 3.3.12 Qualitative studies

Three interview studies explored participant experiences with an e-based physical activity intervention, either in combination with an RCTs (51, 52), or in a pilot study (53).

In the study by Dennett et al. (52), participants of a web-based physiotherapy intervention were interviewed. The study concluded that most participants believed that the web-based PE intervention had increased PA. Key themes were the program's convenience, the value of supervision, and the importance of managing expectations. The program was seen as convenient, flexible, and helpful for sustaining activity levels. Honest discussions about expectations are crucial, especially for those with progressive MS, and web-based interventions are most effective for tech-savvy/competent individuals who prefer flexible, independent exercise (52).

Knox et al. (51) examined perspectives on physiotherapist-supported online vs. paper-based exercise programs. The study identified three key themes from participants with MS: usability, utility, and motivation. Visual and dexterity impairments impacted the program's usability, and participants valued being “pushed” and desired occasional face-to-face contact with physiotherapists. Motivation to exercise varied. Physiotherapists shared similar views. While the online platform was useful, it had limitations, particularly for those with moderate to severe MS, highlighting the need to address these in the growing use of online platforms (51).

The study by Plow et al. (53) explored experiences with a 14-week Wii Fit exercise program. The study found that Wii Fit helped participants engage in exercise. Participants reported that Wii helped build confidence in their abilities to achieve goals related to engagement in leisure activities and remove barriers associated with going to a health club to exercise. However, Wii Fit also reminded participants of their impairments because of its negative feedback and induced initial reactions of intimidations and worries about falling. The study concludes that understanding how to adapt and improve usability of commercially available exergaming could be of benefit to the population with disabling conditions (53).

Exercise for people with MS was facilitated by perceived improvements in physical health, function, and psychosocial wellbeing (98). However, mismatched exercise levels and limited human interaction were barriers. While the exercise program was generally perceived as positive, self-directed exercise remained challenging, emphasizing the need for cost-effective motivational support (98). These insights can guide future strategies to promote sustained exercise participation.

### 3.4 Adherence and drop-out

A total of 22 studies reported on adherence level to the intervention. Seven studies tracked adherence using a diary or logbook, while 11 studies measured adherence automatically as the pwMS logged into the computer or app for the exercise session. Four studies mentioned adherence in their results section but did not detail the measurement methods.

A total of 43 studies provided data on drop-outs, with 14 studies detailing reasons such as pregnancy, relapses, medical issues, health problems, travel or lack of time and unwillingness to participate. In total, 305 pwMS dropped out from a total of 1,974 participants across all studies, resulting in a drop-out rate of 15.4%. Thus, adherence and drop-out analyses in e-based MS exercise studies reveal moderate retention, with drop-outs attributing to a range of challenges.

## 4 Discussion

This scoping review summarized results from 54 studies that delivered at home e-based PE (including 33 RCTs) in pwMS with major differences in study design, and, in general, with a limited number of participants and wide variability in outcome measurements. Notably, e-based PE interventions in RCTs at home showed improvement in depressive symptoms and anxiety. Variation in age and MS disability status (EDSS and PDDS scores) and unevenly matched control groups may explain partly inconsistent results and complicate a synthesis of the findings, evaluation of intervention efficacy on fatigue, walking speed and balance. A homogeneous disability severity among participants in both experimental and control groups may allow more direct comparisons.

A challenge with usual care controls is that we cannot be sure whether all study subjects receive the same usual care, as usual care seem to differ substantially across studies. Moreover, the intervention group may receive a more extensive intervention/more attention which is likely to impact on top of the actual treatment effect.

Adherence to at home, e-based PE is affected by several variables. Most studies had an automatic registration of adherence as the subjects logged into a computer or app while others used recordings from a diary or a logbook. A risk of bias is present for 13 studies that did not report on the frequency of the delivered e-based PE. The assessment of e-based PE at home, such as the intensity and frequency and monitoring of the study participants using new advances in technology, may lead to an increase in adherence (102). This may require supervision, communication and education of the participants. The study with the highest adherence, Van Beek et al. (88), used an app to deliver the intervention, achieving an adherence of 97%. Due to heterogeneity in reporting, it was not clear if the choice of e-based digital platform influenced compliance/adherence. Standardized measures for reporting adherence/drop-out would be valuable for future studies.

In the qualitative studies included in this review the perspectives and experience of pwMS on e-based PE was explored, which led to identification of important factors that influence the motivation and barriers for PE (51–53). Communication and supervision by physiotherapist of the participants about their expectations increased motivation and improved adherence. Limitations of qualitative studies may include a selection bias, e.g. for pwMS who are technologically competent and have self-management. There is a lack of interviews and information from drop-out participants and knowledge about pwMS with severe cognitive impairment who were excluded prior to participation.

Given the variability in symptoms and signs of pwMS and considering heterogeneity of intervention and outcome measurements, there is insufficient evidence to make a recommendation for digital, home-based PE in pwMS. In this review, all RCTs were employed to evaluate outcome, with a wide range of outcome measurements. The studies were generally limited by small sample sizes, absence of measurement of PA prior to intervention, and lack of optimal duration, intensity, and frequency of the intervention. This often led to inconsistent findings and difficulties in comparison of outcomes among studies.

As an example for walking speed, the study by Kahraman et al. (83) reported that walking speed is significantly improved in favor of the at home e-based PE group. However, Conroy et al. (89) and Flachenecker et al. (54), found no significant changes in walking speed between the groups. All these studies were hampered by small sample sizes with a risk for a type 2 error and by short duration of interventions. Furthermore, the experimental groups in comparison to controls had greater disability with predominantly SPMS, and lack of adherence to the PE programme was correlated with a progressive disease course. Furthermore, the study by Kahraman et al. (83) investigated balance and reported an improvement in the intervention group in comparison to a wait-list control group. However, the participants were young and had low EDSS scores which reflect a selection of pwMS (83). Furthermore, they used block sizes of two for the randomization, and this could increase the risk of predictable allocation (83).

Deficits of motor coordination, or ataxia occur often (80%) in pwMS (103), which highlights the importance of measuring ataxia in digital rehabilitation studies of pwMS, in particular as PE intervention is the primary treatment option. However, only one study with 34 pwMS participants assessed ataxia (81) reporting significant improvement in virtual reality-supported task-oriented circuit therapy groups (V-TOCT) as compared to a mobile telerehabilitation (TR) group (81). Secondarily, the increased control of motor coordination may benefit and improve balance and walking parameters.

Cognitive impairment may influence attention, learning, visuospatial abilities, memory, information processing, speed and executive functions, and represents an important cause of disability of pwMS, with an impact on all aspects of quality of life (QoL) (104). A digital, home-based cognitive PE program may be an accessible intervention to improve cognition in pwMS (105). Cognition was investigated in 8 studies with a total of 271 pwMS (57, 61, 66–68, 92, 100). The study by Abasiyanik et al. (67) demonstrated that a pilates-training programme improved cognition in comparison to controls, and Sandroff et al. (100) reported increased SDMT scores in subjects with mild disability in the intervention group. Pagliari et al. (57) reported comparable effects on cognition in the intervention and the control group. And another study by Sebastiao et al. (66) demonstrated no significant changes on cognition between groups. There was no apparent association between the length of the intervention and the number of drop-outs. The differences in outcome may be explained by several factors. The cognitive level at baseline in some studies was high or preserved, and not all common cognitive deficit symptoms were investigated.

Mental health was evaluated in most studies, but with varied methodologies which weaken the conclusions even though the overall trend suggests a beneficial impact on mental health. Approximately 50% of the included studies screened for fatigue which was significantly improved in a few studies (19, 101) in favor of the telerehabilitation group. The study by Flachenecker et al. (54) reported on decreased fatigue in both the intervention group, consisting of internet-based PA promotion, as well as in the control group. However, 3 and 6 months after the end of intervention, fatigue increased in the control group while it was still decreased in the intervention group (54). The authors state that a reason might be that participants in the intervention group continued their PA and exercise after the intervention, but the study failed to measure this (54). Therefore, future studies could consider using accelerometers or other instruments to measure PA after the end of a PE intervention.

The prevalence and burden of depressive symptoms in pwMS underscore the critical importance of efficacious treatments, and PE may be a promising approach for treating depression solely or in combination with antidepressants (28, 106). Significant improvement in symptoms of depression were observed in several studies in the e-based at home intervention group compared to a control group (19, 57, 70, 71, 87, 101). In the study by Kratz et al. (70), only pwMS who met the diagnostic criteria for major depression disorder (MDD) or dysthymia were included in the study. The rest of the studies did not have an inclusion criterion of MDD, participants were not pre-screened for MDD, and nor did they mention the cut-off value for clinically relevant changes in the depression/anxiety instruments used. Significant improvements in symptoms of depression were seen in favor of the e-based at home PE groups, but the outcome measurement instruments, and types of control groups were variable. A longer follow-up post-home-based PE in the studies could add to knowledge on the durability of changes of MDD.

Deterioration of QoL is a hallmark in pwMS and depends on numerous disease-related factors including depression, cognitive deficits, pain, fatigue, as well as disability (107). QoL was investigated in approximately 50% of the studies included this review. In some studies, significant improvements on QoL were reported in favor of the at-home digital PE groups (57, 87, 99). In others, no differences were found (69, 94). Due to variability in outcome measurement instruments and control groups, comparisons between studies are difficult, indicating the complexity of the multifactorial QoL assessment.

Only a few qualitative studies have been performed on e-based at home PE in PwMS (51–53). Exit interviews from pwMS who dropped out, were not performed in any of these studies (51–53). Such information may provide valuable knowledge on how to increase adherence to a PE program and prevent drop-out.

When evaluating the effects of exercise-based interventions, assessment of PA is important. However, the majority of the included studies, 80% (43 studies) did not measure PA. PA was assessed using accelerometry in 20% of the studies (11 studies) and/or used patient-reported measures (i.e. GLTEQ and IPAQ) (19, 61, 64, 72, 73, 75, 85–87, 91, 92, 95, 100, 101). GLTEQ and IPAQ as self-report questionnaires may have recall and social desirability bias. Also, a limited response to questionnaires was observed, leading to further bias in terms of including a selective pwMS group in these studies. The absence of data on comparison between self-report measures and objective measures of PA complicates group comparisons and summaries of outcomes across studies.

The included studies were published between 2008 and 2023 which reflects the increased use of the internet in everyday life (Appendix 1). Of note, an increase in the number of publications on e-based PE interventions in pwMS appeared between 2019 and 2023 compared to previous years. A considerable number of studies did not report on the MS phenotype, disease duration nor the degree of disability. In addition, studies lacked information on the type of PE delivered in the intervention and lacked report on adherence and drop-out. As a result, this scoping review reflects the variability, limitations and difficulties in comparing outcomes of e-based PE. Feasibility studies may be performed before RCTs, reducing the risk of missing data in the main study. Uniform guidelines to standardize data collection and outcome assessment may improve quality of studies.

### 4.1 Limitations of this review study

The heterogeneous objectives, measurement instruments, study designs and control groups across the included studies make comparisons challenging. Likewise, adherence and drop-outs were not uniformly reported. The heterogeneity of the studies, encompassing differences in design, interventions, pwMS populations, control groups, duration of intervention, and outcomes, pose challenges to solid conclusions and interpretation of results.

## 5 Conclusion

Fifty-four studies of pwMS on e-based PE at home were included in this scoping review with different e-based digital platforms, type of rehabilitation programs, and outcome measurement instruments. The studies were limited by small sample size, short follow-up and unevenly matched control groups as to age and degree of disability. Baseline assessment of physical activity and mental status was performed only to a variable degree. Significant improvements in symptoms of depression and anxiety were seen in favor of the e-based at home PE groups, whereas results on fatigue, walking speed and balance were inconsistent. Qualitative studies explored and involved participants' perspectives and experience, which led to increased motivation and adherence. Validation in larger study populations is necessary with proper case ascertainment, uniform assessment of outcome and adherence.

## Data Availability

The original contributions presented in the study are included in the article/Supplementary material, further inquiries can be directed to the corresponding author.
