# Supplementary material for: At home e-based physical exercise programs in patients with multiple sclerosis: a scoping review
Source: Front Neurol. 2024 Oct 15;15:1449519. doi: 10.3389/fneur.2024.1449519 (PMC11518785; doi:10.3389/fneur.2024.1449519)
Supplement: Supplementary file 1 [file Data_Sheet_1.PDF]

Type of e-based platform	Author year	Type of exercise	Length/ frequency	Outcome measurements	Outcome (RCTs) - N/A for non-RCTs	Adherence rate	withdrawal	Funding source/ethics	Adverse events
App	Ehling 2017	Exercises focused on movement, strengthening and coordination of lower limbs and trunk.	12 weeks / daily	Spasticity 0–10 NRS, MI[1], SF-36[2], HADS[3], the Würzburg Fatigue Scale. 11-point normative pain rating scale.	N/A	80%	IG: 1<br>CG: 1	No funding	No report

Dogan 2023	Home-based exercise program including balance, strengthening, coordination, and stretching exercise.	8 weeks / 3 weekly	TIS[4], The kinetic functions sub-parameter of K-ICARS[5], the ABILHAND, MMDT[6], MVN Awinda sensors.	Both the virtual reality supported task-oriented circuit therapy group and the mobile application based telerehabilitation group improved improved upper extremity skills, trunk functions and ataxia symptoms. The first-mentioned group was more effective than the latter in improving the dynamic trunk control and kinetic function.	No report	IG: 2	No report	No report
VanBeek 2020	Dexterity exercises.	4 weeks / 4 weekly	Adherence rate, SUS[7], a Custom User Engagement Questionnaire, 9HPT[8].	N/A	97%	No reports	No report	No report

VanBeek 2022	Dexterity exercises.	4 weeks / 5 weekly	AMSQ[9], the 9HPT[8], CRT[10], the hand-held JAMAR dynamometer, MSIS-29[11].	Tablet app-based dexterity training in MS was not superior compared to theraband virtual reality concerning the arm- and hand function from the participant's perspective. However, tablet app-based dexterity training was found to be effective in improving specific dimensions, namely the fine coordinated finger movements and strength. This effect was apparent compared to a control strengthening exercise program.	92.26%	TAD-MS: 3 Thera-band: 5	This work was supported by the Swiss Multiple Sclerosis Society and Bayer AG.	No report
VanGeel 2020	Walking.	10 weeks / unknown	IPAQ[12], 6MWT[13], T25FW[14], MSWS-12[15], 5-STS[16], 9HPT[8], PASAT[17], SDMT[18], CFI[19], MFIS[20], FSS[21], MSIS-29[11], SF-36[2].	N/A	No report	IG: 3	No report	No report

	Nasseri 2020	Unknown	12 weeks / unknown	EDSS[22], 2MWT[23], 6MWT[13], Timed Tandem Walk Test, 5TSTST[16], Multiple Sclerosis Functional Composite, T25FWT[14], 9HPT[8], SDMT[18], the motor scale of the HAQUAMS[24], MSWS[25], Frenchay activity index, GLTEQ[26].	N/A	No report	CG: 2	No report	No report
Webbased	Donkers 2020	Individually prescribed exercises by a physiotherapist.	6 months / 2 weekly	Exercise adherence, MSIS-29[11], HADS[3], dynamic grip strength and fatigability, T25FWT[14], TUG[27], fall history.	N/A	IG: 38.9(28.1)<br>CG: 34.6(40.8)	IG: 6<br>CG: 3	This study was supported by the Hermes Canada | Multiple Sclerosis Society of Canada Wellness Research Innovation Grant; the Saskatoon Health Region;	No report

							and the College of Medicine, University of Saskatchewan.	
Flachenecker 2020	Individual exercise: strength training & endurance training.	12 weeks / 1-2 weekly	WEIMuS[28], MSIS-29[11]. 2MWT[23], 10MWT[29], Tinetti score.	The positive effects of multimodal inpatient rehabilitation on MS-associated fatigue could be maintained with an individually administered, internet-based physical activity and exercise promotion program for 3 months. In parallel with the reduction of fatigue, HRQoL increased, as well as the walking distance in the 2 min walking test.	Per week mean(SD): 1.7(1.7)	IG: 8 CG: 12	This research was funded in part by the "Freundeskreis Quellenhof e.V.", a nonprofit organization.	No report
Paul 2014	Cardiovascular, strengthening & balance exercises.	12 weeks / 2 weekly	T25FWT[14], BBS[30], TUG[27], MSIS-29[11], Leeds Multiple Sclerosis Quality of Life Scale, Multiple Sclerosis-Related Symptom Checklist, HADS[3]. In addition, to evaluate the feasibility and satisfaction with the webbased physiotherapy programme, participants completed a questionnaire.	N/A	No report	CG: 1	This work was supported by the Chief Scientist Office, Scotland, UK (grant reference CZG/2/528).	3 adverse events unrelated to the intervention.
Conroy 2018	Individualized programmes on individual abilities and expressed goals.	6 months / daily	T25FWT[14], 6MWT[13], BBS[30], MSWS-12[15].	Significant improvement in MSWS-12 scores were seen in patients that were adherent to the internet-based intervention	No report	IG: 10 CG: 17	This work was supported by Merit Review Award I01BX007080 from the US Department of	No report

				programme. The T25FW and 6MWT scores showed no significant worsening or improvement during the six-month study period.			Veterans Affairs Rehabilitation Research and Development Service. This material is the result of work supported with resources and the use of facilities at the VA Maryland Health Care System, Baltimore, Maryland and Johns Hopkins University School of Medicine, Division of Geriatric Medicine and Gerontology Chronic Disease Informatics government.	
Chanpimol 2020	Activities focused on active range of motion, strength, balance, or calisthenic movements.	12 weeks / 3x weekly	T25FWT[14], 2MWT[23], MFIS[20], MSWS-12[15], the Short Physical Performance Battery SPPB.	N/A	58.3%	No report	No funding	No report
Busse 2022	Unknown	6 months / unknown	MFIS[20], UW-SES-SF[31], OxPAQ[32], EQ-5D-5L[33].	N/A	No report	IG: 2 withdrew IG: 3 lost to follow up	This study has been funded by the Multiple Sclerosis Society. The Centre for Trials Research receives	No report

							infrastructure funding from Health and Care Research Wales and Cancer Research UK. HD is supported by the Elizabeth Casson Trust and the NIHR Oxford Health Biomedical Research Centre.	
Paul 2019	Cardiovascular, strengthening and balance exercises.	6 months / 2 weekly	Adherence, 2MWT[23], T25FWT[14], TUG[27], BBS[30], MSIS-29[11], Multiple Sclerosis–Related Symptom Checklist, HADS[3], EQ-5D-5L[33], and steps taken/day measured objectively worn continuously for one week using the activPAL tri-axial accelerometer. To determine the acceptability and feasibility of the study, semi-structured telephone interviews were undertaken with physiotherapists and participants.	N/A	Over 40%	IG: 8<br>CG: 5	Multiple Sclerosis Society, UK (Grant Ref 11).	60 adverse events, not related to the intervention.

Tallner 2016	Strength and endurance training.	6 months / 3 weekly	Health-related quality of life (HrQoL), muscle strength, aerobic capacity and lung function, physical activity, and fatigue (unknown instruments).	The e-training could not influence the primary outcome HrQoL or fatigue, but secondary outcomes, such as muscle strength of the lower extremities, lung function and, especially, physical activity, improved significantly.	36% of participants documented at least 80% of the prescribed sessions.	IG: 10<br>CG: 8	No report	No report
Paul 2010	Individualised exercise programmes.	12 weeks / 2 weekly	T25FWT[14], BBS[30], TUG[27], MSIS-29[11], Leeds Multiple Sclerosis Quality of Life Scale, Multiple Sclerosis-Related Symptom Checklist, HADS[3]. In addition, to evaluate the feasibility and satisfaction with the webbased physiotherapy programme, participants completed a questionnaire.	N/A	No report	CG: 1	This work was supported by the Chief Scientist Office, Scotland, UK (grant reference CZG/2/528).	3 adverse events.
Jeong 2020	Unknown	12 weeks / unknown	The Pittsburg Sleep Quality Index.	N/A	No report	n/a	No report	No report
Finkelstein 2008	Individualised exercise plan with strenghtening, stretching & balance activities.	12 weeks / unknown	T25FWT[14], 6MWT[13], MSWS-12[15], Modified Ashwork Scale, MSQoL-54[34], MSSE[35], Medical Outcome Study Patient Adherence Measure, Client Satisfaction Questionnaire-8,	N/A	No report	n/a	No report	No report

			patient accepteance by use of the attitudinal survey.					
Coulter 2017	Individualised exercises selected based on their baseline assessment and personal goals.	12 weeks / 2 weekly	T25FWT[14], BBS[30], TUG[27], MSIS-29[11], Leeds MS Quality of Life Scale, MS-Related Symptom Checklist, HADS[3]. The intervention group also completed a website evaluation questionnaire and interviews.	N/A	Participants logged on to the website an average 1.3 times per week.	CG: 1	This work was supported by the Chief Scientist Office, Scotland [Grant reference CZG/2/528].	3 adverse events not related to the intervention.
Knox 2022	Exercises for trunk control for independent sitting & transfers.	6.5 months / unknown	In dept interviews face to face and focus group interviews	N/A	No report	17	The Saskatchewan Centre for Patient-Oriented Research.The original trial was funded by a Hermes Canada Multiple Sclerosis Society of Canada Research Innovation Grant.	No report
Pilutti 2013	Lifestyle physical activity, primarily walking.	New content 4 times during the first 8 weeks, 2 times during the final 8 weeks.	GLTEQ[26], ActiGraph accelerometers, FSS[27], MFIS[20], HADS[3], The 15-item short-form McGill Pain Questionnaire, The Pittsburgh Sleep Quality Index, MSIS-29[11], The Patient-Determined Disease Steps.	The study confirms that a lifestyle intervention delivered through the Internet can be effective for increasing everyday physical activity in persons with MS. The intervention group reported significantly less depression, anxiety, and severity of fatigue compared with controls post-trial. The effect for fatigue severity was large in magnitude, whereas the effects for	No report	IG: 4<br>CG: 2	This work was supported, in part, by a grant from the National Multiple Sclerosis Society [PP1695]. LAP was the recipient of a Postdoctoral Fellowship from the Multiple Sclerosis Society of Canada and a Du Pré Grant from the Multiple Sclerosis	No report

					depression and anxiety symptoms were moderate. The lifestyle physical activity intervention had a moderate effect on pain and sleep quality, although these changes did not reach statistically significance.			International Federation.	
	Dennett 2020	Individualized exercise programme.	6 month / 2 weekly	In dept interviews face to face.	N/A	No report	1	No report	No report
Virtual reality	Gutierrez 2013	Postural control and balance training	10 weeks IG: 4 weekly / unknown<br>CG: 2 weekly / unknown	BBS[30], Tinettì scale.	N/A	No report	IG: 1<br>CG: 2	No report	No report
	Pagliari 2021	Unknown	6 weeks / 5 weekly	MSQoL-54[34], MSWS-12[15], BDI[36], FSS[21], Regulatory Emotional Self-Efficacy, State-Trait Anxiety, Box and Block Test, Mini-Balance Evaluation System Test, 9HPT[8], Montreal Cognitive Assessment, SDMT[18], Selective Reminding Test-Long Term Storage, Selective Reminding Test Consistent Long-Term Tetrieval, 10/36 Spatial Recall Test, Paced Auditory Serial Addition Test at 3s, Selective Reminding Test-Delayed Recall, D-10/36-Spatial Recall Test-Delayed Recall, Word List Generation.	Our data support high technological usability as reported by patients, with the majority of participants' evaluations from excellent to best imaginable usability, demonstrating that virtual reality is a relatively suitable telerehabilitation solution. Higher adherence registered in the experimental group. Improving the perception of well-being and self-health can favourably impact pwMS. Beneficial effect observed on mood, with	IG: 86.7% CG: 80.0%	10 in total	This work was supported by the Italian Ministry of Health (Ricerca Corrente and Rete IRCCS Delle Neuroscienze e Della Neuroriabilitazion e – Teleneuroriabilita zione).	No report

					a general reduction of depressive symptoms reported by participants of both groups at post-treatment. Improvements in motor performances associated with balance, postural control, and walking. In total, 63.3% of the telerehabilitation group exhibited improvement in the physical domain of the quality of life. The telerehabilitation group showed greater improvement than the usual care group in Mini-BESTest domains of balance, postural control, and dynamic walking at post-treatment. Higher adherence was registered for telerehabilitation compared with usual care.				
	Kamm 2023	Dexterity exercises.	2 weeks / unknown	Feasibility was measured by the adherence to the protocol, SUS[7], the Custom User Engagement Questionnaire. 9HPT[8], CRT[10], JAMAR dynamometer, AMSQ[9], MSIS-29[11].	N/A	81.8%	no drop-outs	This work was financed by an unrestricted Grant by Roche.	No report
Videoconferencing	Kahraman 2020	Motor imagery training (MIT).	8 weeks / 2weekly	Dynamic Gait Index, T25FW[14], 2MWT[23], TUG[27], the Activities-specific Balance	The telerehabilitation-based imaging training program was feasible	No report	IG: 5<br>CG: 10	No report	No report

			Confidence ABC Scale, a computerized posturography device, postural Stability Test , Limits of Stability Test, the Balance SystemTM SD.	and effective in improving dynamic balance during walking, walking speed and perceived walking ability, balance confidence, cognitive functions, fatigue, anxiety, depression, and quality of life in pwMS.					
Fjeldstad 2016	Unknown		8 weeks / 2 weekly	Functional gait assessment, T25FWT[14], walk across quantifies characteristics of gait as the patient walks across the length of the force plate using the Neurocom Smart Balance Master, BBS[30], SF-36[2], MFIS[20], MSSE[35], EDSS[22], the Activities-Specific Balance Confidence Scale.	N/A	No report	Pt group: 1	No report	No report

Sebastiao 2018	Unknown	12 weeks / 2 weekly – progressed to 5 weekly	T25FW[14], 6MWT[13], TUG[27], SDMT[18], Brief Visuospatial Memory Test, the California Verbal Learning Test, the Short Physical Performance Battery.	N/A	Seven (47%) missed at least one of the biweekly meetings with the exercise trainer.	1 from ig lost to follow-up	This study was supported in part by a pilot grant from the Consortium of Multiple Sclerosis Centers (2016-084666).	
Tarakci 2021	Stretching, strengthening, balance & coordination.	12 weeks / 3 weekly	The Functional Independence Measure, the Nottingham Health Profile, FSS[21], and Quality of Life Scale.	Both approaches led to significant improvements after the intervention. Improvements in the FSS and total score in the NHP were found to be more significant between groups in favor of the supervised exercise approach.	No report	0	The First Author, Ela Tarakci was awarded and received individual funding from Turkish Academy of Sciences as the winner of the Young Scientists Award Program (GEBIP) 2018	No report
Ortiz-Gutierrez 2013	Integrating proprioceptive, visual	10 weeks / 4 weekly	Composite Equilibrium Score, Visual Preference Ratio,	N/A	No report	IG: 1<br>CG: 2	No report	No report

		& vestibular sensory information.		Vestibular Ratio, Visual Ratio, Somatosensory Ratio.					
E-mail	Keytsman 2019	Bicycling.	6 months / 3weekly	Weight, BMI, fat mass, fat percentage, total fat, VO2 max, workload, heart rate max, heart rate recovery, lactat max, lactat peak, respiratory exchange ratio.	N/A	IG: ~95% HC: ~90%	IG: 5 HC: 3	No report	No report
	Kratz 2014	Unknown	12 weeks / 8 sessions	The 7-Day Physical Activity Recall Interview, HAM-D[37], The Positive and Negative Affect Schedule.	Motivational interviewing intervention was related to decreased depressive symptoms by way of changes in a number of specific mechanistic pathways. The intervention results in increased physical activity compared with the waitlist control. Physical activity was related to increased positive affect, related to lower depressive symptoms.	No report	IG: 2 CG: 9	No report	No report

Abasiyanik 2018	Pilates.	Once a week for 8 weeks plus a two-day home exercise program.	6MWT[13], T25FWT[14], TUG[27], MSWS-12[15], The Biodex Balance System, The Falls Efficacy Scale International, The Activities-Specific Balance Confidence Scale, The Curl-Up Test, Maximum inspiratory and expiratory pressure, The Brief International Cognitive Assessment for Multiple Sclerosis.	Our results demonstrated that an 8-week Clinical Pilates training program can improve walking, balance, respiratory functions, and cognitive functions and decrease fall risk in pwMS.	No report	IG: 5<br>CG: 4	The Multiple Sclerosis Research Association funded this study in terms of purchasing the manovacuometer (Medical Electronic Construction, Pocket-Spiro MPM100, Brussels, Belgium).	No report
Bombardier 2013	Unknown	12 weeks / unknown	HAM–D[37].	The treatment group evidenced significantly lower depression severity. Physical activity increased significantly more in the treatment condition, though it did not mediate improvement in depression severity.	No report	IG: 22<br>CG: 9	No report	No report
Kratz 2020	Cycling, treadmill overground walking, lower extremity strength training.	8 weeks / 3 weekly	Feasibility, acceptability, Ecological Momentary Assessment Fatigue Intensity and Fatigue Interference, FSS[21].	N/A	IG: 7.6 sessions out of 8.<br>CG: 7.8 sessions out of 8	IG: 1	No report	

Turner 2016	Unknown	6 months<br>7 times during the first 2 months, 4 times during the second 2 months, and twice during the final 2 months.	The Depression Module of the Patient Health Questionnaire-9, MFIS[20].	Brief telephone counseling and telehealth home monitoring to promote physical activity (TC) can improve fatigue among individuals with MS compared to self-directed education (EC). By 6-month follow-up, participants in the TC condition experienced significant reductions in fatigue relative to individuals in EC. For 33.3% of participants in TC, this reduction was clinically significant	No report	IG: 1	No report	No adverse events.

Combination	Dlugonski 2012	Unknown	12 weeks / unknown	GLTEQ[26], MSWS-12[25], MSIS-29[11], PDDS[38], process evaluation questionnaire.	An Internet-delivered behavioral intervention for persons with MS resulted in a large increase in physical activity that was sustained following the intervention, lager for the intervention group. Non-significant condition-by-time interactions for walking mobility and HRQOL. No significant correlations between change in physical activity and HRQOL or walking mobility change from pre- to post-trial within the intervention and control groups.	77%	11	No report	No report
	Mardaniyan Ghahfarrokhi 2021	Range of motion, balance exercises, posture exercises, strength exercises, pelvic control	8 weeks / 3 weekly	Age, height, weight, body mass index, fat mass, lean mass, EDSS[22], MS duration, SDMT[18], 10MWT[29], T25FWT[14], TUG[27], the Six Spot Step Test,	N/A	First 4 weeks HBNFT group: 92.2% HBRT group: 91.1%	no drop-outs	No report	No report

	exercises and torso stability.		6MWT[13], 5TSTS[16], self-reported feasibility questionnaire.		Second 4 weeks:<br>HBNFT group: 96.7%<br>HBRT group: 95.6%.			
Sandroff 2014	Unknown	6 months / unknown	SDMT[18], 6MWT[13], IPAQ[12], PDDS[38].	SDMT scores increased in the intervention condition as a function of disability. 6MW distance increased in the intervention condition, and this did not occur as a function of disability. Greater increases in physical activity were associated with faster CPS and better walking performance in the intervention condition.	IG: 88.6 %	IG: 4<br>CG: 2	No report	No report

Videoconferencing & telephone	Plow 2022	Pedometer-based walking programme as well as another unknown training.	12 weeks / unknown	FIS[39], GLTEQ[26], MSIS-29[11], The Energy Conservation Strategy Survey.	No interaction terms were statistically significant in the moderation analysis. However, the responder analysis showed that baseline psychosocial characteristics and mental function were significantly different ($p < 0.05$) between responders and non-responders. Specifically, non-responders on the FIS at post-test in the PA-only intervention had significantly lower baseline scores in goal setting for engaging in fatigue self-management behaviours. Also, nonresponders on the GLTEQ at post-test in the FM+ intervention had significantly worse baseline scores in mental function.	No report	n/a	This work was supported through the National Multiple Sclerosis Society (NMSS, Grant # RG4665-A-1).	No report
DVD & telephone	Fleming 2019	Pilates.	8 weeks / 2 weekly	MFIS[20], Profile of Mood States Brief Form, The State Trait Anxiety Inventory, HADS[3], Quick Inventory of Depressive Symptomatology, Seven-day	N/A	No report	IG: 2	No report	No report

Videoconferencing or phone & newsletter & fitbit one (to measure physical activity)	Manns 2020	Weekly coaching sessions encouraged breaking up prolonged sitting bouts (focus of the SitLess stage) or promoting increased steps per day in addition to interrupting sitting.	15 weeks / unknown	Physical Activity Recall Scale, GLTEQ[26]. ActivPAL3.	N/A	No report	5	No report	15 adverse events in 10 participant regarding falls not associated with the intervention.
Google meet & Zoom & Instagram	Najafi 2023	Yoga & pilates.	8 weeks / 3 weekly	Cortisol- and prolactin serum levels, MSQoL-54[34], General Health Questionnaire, IPAQ[12], IBD[36], T25FW[14], MSQoL-54[34].	The investigation results after eight weeks of tele-Pilates and tele-yoga in females with MS showed an increase in prolactin serum levels; improvements in depression; and the enhancement of physical activity levels, walking speed, and QoL.	Tele-pilates: 5<br>Tele-yoga: 4<br>CG: 2	n/a	No report	No report
Website & Yamax SW-401	Pilutti 2014	Lifestyle physical activity, primarily walking.	6 months<br>7 times during the first 2	PDDS[38], BMI, Whole-body bone mineral content, bone mineral density and soft tissue	We provide preliminary evidence that an internet-delivered life-	No report	IG: 6<br>CG: 4	No report	No report

Digiwalker pedometer & Goal Tracker & Skype	months, 4 times during the second 2 months, and twice during the final 2 months.	composition, GLTEQ[26], ActiGraph model GT3X accelerometers.	style physical activity intervention might improve bone health and body composition, assessed using criterion standard measurement, in persons with MS.

DVD & programme manual & calendars & logbook & behavioural change interaction & videochats & newsletter	Learmonth 2016	Resistance and aerobic training.	16 weeks / 2 weekly	Feasibility was assessed in the domains of process (e.g., recruitment), resource (e.g., monetary costs), management (e.g., personnel time requirements) and scientific outcomes (e.g., treatment effect).	N/A	90%	4 from IG, 2 from CG	No report	No report
Telephone & teleconferencing	Plow 2019	Walking.	PA group: 3 teleconference sessions followed by 4 individually	FIS39, GLTEQ[26], Accelerometers (ActiGraph triaxis accelerometers), MSIS-29[11], treatment fidelity and possible confounders (tracking	Group teleconferences followed by tailored phone calls have a small yet statistically significant effect in	>95%	n/a	No report	No report

			tailored phone calls. The FM+ and CC groups consisted of 6 group teleconference sessions followed by 4 individually tailored phone calls.	attendance and monitoring the delivery of the interventions).	promoting physical activity and reducing fatigue impact in people with MS.				
Other									
Home-care activity desk (H-CAD)	Huijgen 2008	Upper limb functional exercises.	4 weeks / 5 weekly	The Action Research Arm Test, 9HPT[8].	Based on the high satisfaction of both patients and therapists, together with the finding that the HCAD training is as feasible as usual care when comparing their clinical outcomes, the HCAD intervention might be valuable as treatment in future care for stroke, TBI and MS patients living at home to train their arm/hand function.	No report	IG: 7<br>CG: 4	No report	No report

Home Automated Telemanagement (HAT)	Jeong 2021	Individualised exercises.	12 weeks / unknown	MSQOL-54[34].	Physical telerehabilitation in the intervention group resulted in more prominent improvements in QOL and symptom reduction as compared to a usual care control group. More than 70% of the patients in the intervention group showed improvements in sub-scales reflecting physical health, role limitations emotional, health perceptions, cognitive function, change in health and satisfaction with sexual function, and more than 60% of these patients improved in scores of role limitations-physical, social function, health distress, sexual function, and overall quality of life. In particular, the use of the physical telerehabilitation system showed statistical differences in major MS symptoms including pain and cognitive function with significant changes in	No report	n/a	No report	No report

					the corresponding sub-scale scores (pain: 27.3%, cognitive function: 35.3%).				
Nintendo Wii	Prosperini 2012	Balance exercises.	12 weeks intervention, 12 weeks observation / 5 times weekly.	Force platform–based measures of static standing balance, the 4-Step Square Test, T25FWT[14], MSIS-29[11]. Self-reported number of accidental falls	N/A	No report	IG: 1<br>CG:1	No report	5 adverse events.

				(defined as an unexpected contact of any part of the body with the ground) that occurred in the 12-week period before randomization was also asked of each patient. Force platform–based measures of static standing balance were collected with a monoaxial force platform.					
Nintendo Wii	Thomas 2017	Unknown	12 months for the immediate group and for about 6 months for the delayed group.	GLTEQ[26], activPAL3 triaxial accelerometer, HADS[3], EQ-5D-5L[33], MSIS-29[11], the Fatigue Symptom Inventory, SF-36[2], the Spinal Cord Injury Exercise Self-Efficacy Scale, MSSE[35], 2MWT[23], Step Test, Steady Stance Test, the Instrumented Timed Up and Go Test, Gait Stride-time Rhythmicity, Static Posturography, 9HPT[8].	N/A	The Wii was used on around 30% of days during the first 6 months and 19% of days in the second 6 months,	IG: 2	This study was funded by a project grant awarded by the MS Society in the UK (ref no. 933/10). It is included in the National Institute of Health Research Clinical Research Network (NIHR CRN) portfolio (ID 13130).	No report
Nintendo Wii	Prosperin i 2014	Balance exercises.	12 weeks / 5 weekly	Static posturography, brain MRI scan.	N/A	No report	IG: 5<br>CG: 4	no report	No report
Preloaded tablet	Kim 2022	Yoga, pilates & neurorehabilitation activities.	First 8 weeks: 60 min 2 times weekly<br>next 4 weeks: only yoga and pilates once a week.	Unknown	N/A	No report	N/A	The work of this article was supported by the Patient-Centered Outcomes Research Institute (PCORI), United	No report

								States (Award #MS-1511-33653).	
Home-care activity desk (H-CAD)	Hermens 2008	Exercises of the upper limb for reaching, grasping, lateral pinch, pinch grip, holding, manipulation & finger dexterity.	4 weeks / 3 min / day / 5 days /week	The Action Research Arm Test, the Wolf Motor Function Test, the Barthel Index, 9HPT[8], SF-36[2].	In general, we can conclude that the arm/hand function remained at the same level in both groups	No report	IG: 9 CG: 6	No report	No report
Nintendo Wii	Plow 2014	Unknown	14 weeks / unknown	Unknown	N/A	No report	n/a	this study was supported by a grant from the consortium of multiple sclerosis centers (CMSC) and the national multiple sclerosis society (NMSS) post-doctoral training grant.	No report
Nintendo Wii	Plow 2011	Unknown	14 weeks / unknown	Unknown	N/A		n/a	No report	This study was supported by a grant from the consortium of multiple sclerosis centers (CMSC) and the national multiple sclerosis society (NMSS) post-doctoral training grant.

[1] Motricity Index

[2] The Short-Form-36 Health Survey

[3] The Hospital Anxiety and Depression Scale

[4] The Trunk Impairment Scale

[5] The International Cooperative Ataxia Rating Scale

[6] The Minnesota Manual Dexterity Test

[7] The System Usability Scale

[8] The Nine-Hole Peg Test

[9] The Arm Function in Multiple Sclerosis Questionnaire

[10] The Coin Rotation Task

[11] The Multiple Sclerosis Impact Scale-29

[12] The International Physical Activity Questionnaire

[13] The 6 Minute Walk Test

[14] The Timed 25-foot Walk Test

[15] The Multiple Sclerosis Walking Scale-12

[16] The 5-Repitition Sit-to-Stand Test

[17] The Paced Auditory Serial Addition Test

[18] The Symbol Digits Modalities Test

[19] The Cognitive Fatigability Index

[20] The Modified Fatigue Impact Scale

[21] The Fatigue Severity Scale

[22] Expanded Disability Status Scale

[23] The 2 Minute Walk Test

[24] The Hamburg Quality of Life Questionnaire Multiple Sclerosis

[25] The Multiple Sclerosis Walking Scale

[26] The Godin–Leisure Time Exercise Questionnaire

[27] The Timed Up and Go Test

[28] The Würzburg Fatigue Inventory for Multiple Sclerosis

[29] The 10 Meter Walk Test

[30] The Berg Balance Scale

[31] The University of Washington 6-Item Short Form Self-Efficacy Scale

[32] The Oxford Participation and Activities Questionnaire

[33] The Five-Level EuroQol-5 Dimensions Health State Utility Scale

[34] The Multiple Sclerosis Quality of Life-54 Scale

[35] The Multiple Sclerosis Self-Efficacy Scale

[36] The Beck Depression Inventory

[37] The Hamilton Depression Rating Scale

[38] The Patient-Determined Disease Steps Scale

[39] The Fatigue Impact Scale
