# Supplementary material for: At home e-based physical exercise programs in patients with multiple sclerosis: a scoping review
Source: Front Neurol. 2024 Oct 15;15:1449519. doi: 10.3389/fneur.2024.1449519 (PMC11518785; doi:10.3389/fneur.2024.1449519)
Supplement: Supplementary file 2 [file Data_Sheet_2.PDF]

Type of e-based platform	Author year	Study Design, Country	Type of control group	Number of participants<br>Intervention group<br>Control group	Time of diagnosis<br>Intervention group<br>Control group	EDSS/PDDS<br>Intervention group<br>Control group	Age<br>Intervention group<br>Control group
App	Ehling 2017	Prospective pilot trial, Austria	Screenshots of selected exercises and patient reported outcomes on hard copy (paper). Wait-list for the APP-intervention.	5 m / 5 f<br>6 m / 4 f	Mean: 12.6 (8.8-16.5)<br>Mean: 16.3 (10.6-22.0)	Mean EDDS: 4.2 (3.1-5.3)<br>Mean: EDDS: 5.4 (4.2-6.5)	Median: 46.6<br>Median: 50.5

Dogan 2023	Randomzied controlled trial, Turkey	Virtual reality supported task-oriented circuit training in the clinic	2 m / 13 f<br><br>6 m / 11 f	Unknown	Mean (SD): EDSS 3.74 ± 0.92<br><br>Mean: EDSS 3.94 ± 1.04	Mean: 36 ± 8.19<br><br>Mean: 38.76 ± 5.53
VanBeek 2020	Pilot feasibility interventional study, Switzerland	N/A	0 m / 9 f	Mean: 10.56 ± 9.76	Mean: EDSS 3.89 ± 1.95	53.89 ± 12.27

vanBeek 2022	Randomzied controlled trial, Switzerland	arm- and hand strengthening exercises using Thera-band	5 m / 21 f<br>7 m / 15 f	Mean: 12.33 ± 8.23<br>Mean: 11.69 ± 9.57	Mean: EDSS: 2.98 ± 1.81<br>Mean: EDSS: 3.03 ± 2.23	Mean: 50.84 ± 14.84<br>Mean: 48.40±14.61
VanGeel 2020	Clinical trial, Belgium	N/A	12 f	Median: 7 years	Unknown	Median: 42.5

	Nasseri 2020	Pilot randomized controlled trial, Germany	In the control group, participants received a leaflet with unspecific information about exercising in general.	10 m / 9 f<br>9 m / 11 f	Mean: 13.1 ± 5.6.<br>Mean: 20.1 ± 13.0	Median EDSS: 3.5 (2.0-6.0.<br>Median: EDDS 3.5 (3.0-6.0)	Mean: 49.6 ± 8.5.<br>Mean: 52.5 ± 7.3
Webbased	Donkers 2020	Randomised controlled pilot trial, Canada	Written, home-based exercise program.	12 m / 20 f<br>5 m / 11 f	Mean: 20.0 ± 11.3<br>Mean: 18.4 ± 10.7	Mean: PDDS=4.2 ± 1.6<br>Mean: PDDS=4.8 ± 1.7	Mean: 54.6±11.9<br>Mean: 53.8 ± 12.2
	Flachenecker 2020	Randomised, single blind conrolled study, Germany	usual care control group	12 m / 22 f<br>13 m / 17 f	Mean: 13.4 ± 7.9<br>Mean: 9.0 ± 7.5	Median: EDSS 4.3 (3.5–5.0)<br>Median: EDSS 4.0 (3.0–6.0)	Mean: 47.6 ± 9.2<br>Mean: 46.4 ± 12.2

Paul 2014	Randomized controlled pilot trial, UK	usual care which was general advice on exercise and signposting to local exercise options.	3 m / 12 f<br>3 m / 12 f	Mean: 12.5 ± 7.1<br>Mean: 12.8 ± 10.9	Mean: EDSS: 6.0 ± 0.5<br>Mean: EDSS 5.8 ± 0.5	Mean: 50.8 ± 7.4<br>Mean: 52.5 ± 14.3
Conroy 2018	Randomised, single-blind controlled trial, USA	Routine home-based exercise - The control group received individualised exercise pre-scriptions in paper hand-out form common for PT home exercise programmes.	9 m / 7 f<br>3 m / 5 f	Mean: 14.2 ± 7.8<br>Mean: 14.7 ± 9.8	Mean: PDDS 4.5 ± 1.6<br>Mean: PDDS 3.9 ± 1.4	Mean: 50.4 ± 8.1<br>Mean: 54.3 ± 5.9
Chanpimol 2020	Pilot study, USA	N/A	2 m / 8 f	Mean: 8.0 ± 6.3	Median: EDSS 5.0 (3.5-6.0)	Mean: 49.6 ± 9.0
Busse 2022	Single-arm non-randomized feasibility study, UK	N/A	6 m / 15 f	Mean: 17.7 ± 11.7	Range: EDSS 6-7.5	Mean: 60.9 ± 9.2
Paul 2019	Multicentre, randomized controlled feasibility study, UK	printed sheet of exercises.	13 m / 32 f<br>8 m / 37 f	Median: 10 (6-18)<br>Median: 15 (10-23)	Median EDSS: 6.0 (6-6)<br>Median: EDSS 6.0 (6-6)	Mean: 55.6 ± 10.2<br>Mean: 56.5 ± 9.1

Tallner 2016	Randomzied controlled trial, Germany	wait-list control	15 m / 44 f<br>17 m / 50 f		Mean: 9.8 ± 9.2<br>Mean: 9.2 ± 7.2	Mean(SD): EDSS 2.8 ± 0.8<br>Mean: EDSS 2.7 ± 0.8	Mean: 40.9 ± 10.4<br>Mean: 40.7 ± 9.5
Paul 2010	Randomized controlled pilot trial, Scotland	usual care which was general advice on exercise and signposting to local exercise options	3 m / 12 f<br>3 m / 12 f		Mean: 12.5 ± 7.1<br>Mean: 12.8 ± 10.9	Mean: EDSS 6.0 ± 0.5<br>Mean: EDSS 5.8 ± 0.5	Mean: 50.8 ± 7.4<br>Mean: 52.5 ± 14.3
Jeong 2020	Longitudinal cohort study, USA	N/A	2 m / 15 f		Range between 2.5 to 50.8 years	Unknown	Mean: 60.1 ± 11.4
Finkelstein 2008	Pilot study, USA	N/A	2 m / 10 f		Mean: 13 ± 7	Mean: PDDS 3.7 ± 1.1	Mean: 52 ± 4
Coulter 2017	Randomised controlled pilot study, Scotland	usual care which was general advice on exercise and signposting to local exercise options outwith	3 m / 12 f<br>3 m / 12 f		Mean: 12.5 ± 7.1.<br>Mean: 12.8 ± 10.9	Mean: EDSS 6.0 ± 0.5<br>Mean: EDSS 5.8 ± 0.5	Mean: 50.8 ± 7.4.<br>Mean: 52.5 ± 14.3

			the health service.				
	Knox 2022	Qualitative study, UK	paper based exercise	13<br>5	Median: 19 years	Median: PDSS 4 (2-6)	Median: 57.5
	Pilutti 2013	Randomzied controlled trial, USA	wait-list control	30 f / 11 m<br>32 f / 9 m	Mean: 10.6 ± 7.1)<br>Mean: 13.0 ± 9.1	Median: PDDS 2.0<br>Median: PDDS 3.0	Mean: 48.4 ± 9.1<br>Mean: 49.5 ± 9.2
	Dennett 2020	Qualitative study, UK	paper based exercise	1 m / 10 f	Range 1-40 years	Range EDSS 4-6.5	Range 28-68
Virtual reality	Gutierrez 2013	Clinical trial, Spain	The control group (CG; *n* = 25) received physiother- apy treatment twice a week (40 min per session) at the MS Madrid Association and Foundation. The treatment was based on low-loads strength exercises (10 min per session), propioception exercises on unstable surfaces and gait facilitation exercises (20 min per session), and,	11 m / 13 f<br>9 m / 14 f	Mean: 9.68 ± 6.76<br>Mean: 10.86 ± 5.40	Percentage: EDDS score 3 (16.4%) EDDS 4 (75.5%) EDDS 5 (8.1%)<br>Percentage: EDDS score 3 (21.7%), EDDS 4 (60.9%) EDDS 5 (17.4%)	Mean: 39.69 ± 8.13<br>Mean: 42.78 ± 7.38

		finally, muscle-tendon stretching (10 min per session), (Patti et al., 2002).					
Pagliari 2021	Multicentre, rater-blinded, active controlled, and randomized trial, Italy	Usual care group who received at-home conventional motor and cognitive rehabilitation. the UC was an active comparator treat- ment and consisted of a written home-based self-administrated booklet with conventional motor and cogni- tive exercises tailored for pwMS with the same intensity and duration of the TR treatment.	12 m / 18 f<br>12 m / 18 f		Mean: 12.68 ± 6.72<br>Mean: 15.36 ± 7.17	Median: EDSS 5.0 (3.50–6.0).<br>Median: EDSS 4.50 (3.50–6.0)	Mean: 48.33 ± 9.66<br>Mean: 52.23 ± 9.34
Kamm 2023	Single-center, single-arm observational study, Switzerland	n/a	4 m / 7 f		Mean: 15.38 ± 9.95	Mean: EDSS 4.28 ± 1.48	Mean: 49.20 ± 10.87

Videoconferencing	Kahraman 2020	Randomzied controlled trial, Turkey	CG (waitlist-group)+HC.	4 m / 16 f<br>1 m / 14 f<br>HC: 6 m / 14 f	Median: 4<br>Median: 4	Median: EDSS 1(0-1.75)<br>Median: EDSS 2 (0-2.5)	Median: 34.5<br>Median: 31
	Fjeldstad 2016	Randomized feasibility and efficacy pilot study, USA	Group 1-unsupervised home exercise programme (control group)	3 m / 7 f<br>PT=2 m / 7 f<br>CG=4 m / 6 f	Unknown	Median: EDSS TR group=4.4 ± 1.0. PT group=4.3 ± 1.4.<br>Mean: EDSS 4.4 ± 1.1	Mean: TR group 55.1 ± 13.9,<br>PT group: 54.7 ± 13.5.<br>Mean: CG 54.4 ± 10.8
	Sebastiao 2018	Feasibility randomized controlled trial, USA	a light intensity stretching and minimal muscle strengthening program based on an illustrated manual for persons with MS developed by the National Multiple Sclerosis Society that involves major muscle groups of the upper and lower body.	2 m / 13 f<br>1 m / 9 f	Mean: 21.9 ± 10.7<br>Mean: 19.9 ± 11.2	Median: EDSS 3.75<br>EDSS: 4.25	Mean: 63.8 ± 4.1<br>Mean: 65.1 ± 5.2

Tarakci 2021	Single-blind randomized controlled trial, Turkey	N/A	4 m / 11 f<br><br>3 m / 12 f		Mean: 8.86 ± 4.50<br><br>Mean: 6.20 ± 3.96	Median: EDSS 3.46 ± 1.31<br><br>Median: EDSS 3.40 ± 1.53	Mean: 39.46 ± 10.59<br><br>Mean: 41 ± 11.09
Ortiz-Gutierrez 2013	Preliminary interventional study, Spain	The control group (CG; *n* = 25) received physiotherapy treatment twice a week (40 min per session) at the MS Madrid Association and Foundation. The treatment was based on low-loads strength exercises (10 min per session), propioception exercises on unstable surfaces and gait facilitation exercises (20 min per session), and, finally, muscle-tendon stretching (10 min per session).	11 m / 13 f<br><br>9 m / 14 f		Mean: 9.68 ± 6.76.<br><br>Mean: 10.86 ± 5.40	Experimental group: Score 3 EDSS 16.4 % (N = 4) Score 4 EDSS 75.5 % (N = 17) Score 5 EDSS 8.1 % (N = 3)	Mean: 39.69±8.13<br><br>Mean: 42.78 ± 7.38

E-mail	Keytsman 2019	Clinical trial, Belgium	Healthy controls	12 m / 6 f<br>14 m / 5 f	Unknown	Mean: EDSS 1.9 ± 1.1	Mean: 41.7±8<br>Mean: 41.5 ± 9.9
	Kratz 2014	Two-group, parallel randomized controlled trial, USA	wait-list control group	5 m / 39 f<br>8 m / 40 f	Mean: 9.4 ± 7.1,<br>Mean: 11.7 ± 8.3	Number (%): EDSS 4.0: 24 (54), EDSS 4.5-6.5: 20(6)<br>Number (%): EDSS 4.0: 24(50), EDSS 4.5-6.5: 24(50)	Mean: 47.1±8.9<br>Mean: 49.7 ± 7.9

Abasiyanik 2018	Randomzied controlled trial, Turkey	home exercise: The control group subjects were given written standardized home exercises for 3 times per week for 8 weeks.	4 m / 12 f<br>6 m / 11 f	Mean: 12.59 ± 6.23<br>Mean: 9.83 ± 8.7	Mean: EDDS 3.06 ± 1.6<br>Mean: EDDS 3.24 ± 1.7	Mean: 42.50 ± 6.76<br>Mean: 48.24 ± 11.79
Bombardier 2013	Single-blind, two-arm randomized controlled trial, USA	waitlist control	70 (75.7% female)<br>60 (80% female)	Mean: 6.3<br>Mean: 6.4	Unknown	Mean: 47.5<br>Mean: 45
Kratz 2020	Pilot randomized study, USA	in person with trainer 1/week	1 m / 9 f<br>1 m / 9 f	Median: 13.5<br>Median: 4.5	Median: PDDS 2<br>Median PDDS 3.5	Mean: 45.9 ± 8<br>Mean: 50.7 ± SD 7.4
Turner 2016	Single blinded rct, USA	self directed education	9 m / 22 f<br>14 m / 19 f	Mean: 11.3 ± 9<br>Mean: 11.8 ± 10.4	Mean: Mobility performance scale: 2.3 ± 1.5	Mean: 52.7 ± 11.6

							Mean: Mobility performance scale 2.73 ± 1.35	Mean: 53.6 ± 13.1
Combination	Dlugonski 2012	Randomzied controlled trial, USA	Wait-list control	4 m / 18 f		Mean: 10.3 ± 9.2	Median: PDDS 1.0	Mean: 48.5 ± 10.1
				2 m / 21 f		Mean: 8.5 ± 6.2	Median: PDDS 1.0	Mean: 44.8 ± 9.1

Mardaniyan Ghahfarrokhi 2021	Pilot randomized feasibility study, Iran	home-based neuro-functional training	9 m / 3 f<br><br>11 m / 2 f	Mean: 9.73 ± 5.29<br><br>Mean: 8.30 ± 6.48	Mean: EDSS 4.13 ± 0.97<br><br>Mean: EDSS 4.57 ± 1.30	Mean: 39.87 ± 9.09<br><br>Mean: 37.50 ± 8.58
Sandroff 2014	Randomzied controlled trial, USA	wait-list control	PDDS 0-2: 6 m / 12 f<br><br>PDDS 3-6; 4 m / 15 f<br><br>PDDS 0-2; 3 m / 15 f<br><br>PDDS 3-6: 6 m / 15 f	Mean: PDDS 0-2: 9.0 ± 7.2, PDDS 3-6: 12.3 ± 6.3<br><br>Mean: PDDS 0-2: 12.7 ± 9.7, PDDS 3-6: 14.0 ± 9.0	PDSS 0-2: n=18) PDSS 3-6 (n=19)<br><br>PDSS 0-2 (n=18) PDSS 3-6(n=21)	Mean: PDDS 0-2; 45.1 ± 10.1, PDDS 3-6; 52.1 ± 6.4<br><br>Mean: PDDS 0-2; 49.0 ± 10.0, PDDS 3-6; 51.6 ± 6.7

Videoconferencing & telephone	Plow 2022	Randomzied controlled trial, USA	Participants (n 1⁄4 208) were randomly assigned into a contact- control intervention (CC), physical activity-only inter- vention (PA-only), or physical activity plus fatigue self-management intervention (FMþ) The PA-only intervention consisted of three group teleconfer- ence sessions delivered once per week. The FMþ and CC interventions consisted of six group teleconferencesessions delivered once per week. After the third ses- sion, participants in each group received four individ- ually tailored phone	11 m / 46 f<br>PA-only: 9 m / 41 f<br>FM+: 5 m / 58 f	All patient groups: Mean number of years 12.67 ± 8.68	Unknown	Mean: PA: 51.42 ± 9.85, FM+ 53.06 ± 6.58<br>Mean: 52.09 ± 8.83

calls delivered once every other week. Participants in the PA-only and FMþ interven- tions during the group teleconference sessions were taught how to engage in a pedometer- based walking programme, set goals, overcome obstacles, and self- monitor progress. Participants in the FMþ interven- tion received additional content adapted from the Managing Fatigue programme.13,3 0 Participants in the CC intervention received generic health information (e.g. healthy eating and

			preventive screening).				
DVD & telephone	Fleming 2019	Randomized controlled pilot trial, Ireland	supervised pilates session or wait-list control	0 m / 6 f<br><br>supervised: 0 m / 5 f<br><br>wait-list: 1 m / 6 f	Unknown	Unknown	Mean: 46.0 ± 9.4,<br><br>Mean: Supervised group:

							53.8 ± 7.95,<br><br>Wait-list group: 51.3 ± 6.8
Videoconferencing or phone & newsletter & fitbit one (to measure physical activity)	Manns 2020	Pre-post, single-group, repeated measures intervention design, Canada	N/A	2 m / 37 f	Mean: 14.3 ± 11.3	Median: EDSS 5.5	Mean: 50.5 ± 10.3
Google meet & Zoom & Instagram	Najafi 2023	Single-blinded RCT, Iran	female MS patients maintained their current lifestyle without regular physical activity or exercise for eight weeks	Tele pilates: 15 f<br><br>Tele-yoga 15 f	Mean: Tele-pilates: 10.93 ± 4.38, Tele-yoga 8.0 ± 5.84<br><br>Mean: 9.27 ± 8.37	Mean: EDSS Tele-pilates: 2.5 ± 1.32,<br><br>Tele-yoga: 2.5 ± 1.19<br><br>Mean: EDSS 2.66 ± 1.03	Mean: Tele-pilates: 36.20 ± 4.33, Tele-yoga: 37.40 ± 6.03

								Mean: 40.40 ± 5.35
Website & Yamax SW-401 Digiwalker pedometer & Goal Tracker & Skype	Pilutti 2014	Secondary analysis of data from a randomized controlled trial, USA	waitlist control	11 m / 30 f<br>9 m / 32 f		Mean: 10.67 ± 1<br>Mean: 13.09 ± 1	Median: PDDS 2.0<br>Median PDSS 3.0	Mean: 48.4 ± 9.1<br>Mean: 49.5 ± 9.2

DVD & programme manual & calendars & logbook & behavioural change interaction & videochats & newsletter	Learmonth 2016	Feasibility randomized controlled study, USA	wait-list control	1 m / 28 f<br><br>1 m / 27 f	Mean: 14.8 ± 8.5.<br><br>Mean: 13.0 ± 7.7	Median: EDSS 1.25<br><br>Median EDSS 2	Mean: 48.7 ± 10.4<br><br>Mean: 48.2 ± 9.1
Telephone & teleconferencing	Plow 2019	Randomzied controlled trial, USA	Participants in the CC intervention received information on	pa-only: 14 m / 55 f<br><br>FM+: 7 m / 63 f<br><br>CC: 11 m / 58 f	Mean: PA-only: 14.1 ± 9.6<br><br>Mean: FM: 12.7 ± 7.9,	Number (%): PDSS: Mild disability PA: 13 (18.8) FM+: 9 (12.9) CC: 12	Mean: PA-only=51.2 ± 9.2, FM+=53.2 ± 6.5,

			health topics relevant to MS.		Mean: cc: 11.4 ± 8.1	(17.4)<br>Moderate disability PA: 18 (26.1) FM+: 13 (18.6) CC: 10 (14.5)<br>Gait disability PA: 17 (24.6) FM+: 21 (30.0) CC: 24 (34.8)<br>Early cane PA: 14 (20.3) FM+17 (24.3) CC: 14 (20.3)<br>Late cane PA: 7 (10.1) FM+10 (14.3) CC: 9 (13.0)<br><br>PDSS number(%):<br>Mild disability CC: 12 (17.4)<br>Moderate disability CC: 10 (14.5)<br>Gait disability CC: 24 (34.8)<br>Early cane CC: 14 (20.3)<br>Late cane CC: 9 (13.0)	Mean: 51.8 ± 9.3
Other							
Home-care activity	Huijgen 2008	Randomized controlled multicentre trial,	Subjects in the control group received usual	11 m / 13 f	Mean 15.1 ± 8.6.	Unknown	Mean: 48 ± 12.

desk (H-CAD)		Italy, Spain & Belgium	care and generic exercises prescribed by their physicians.	7 m / 4 f	Mean: 10.2 ± 7.6		Mean: 51 ± 14
Home Automated Telemanagement (HAT)	Jeong 2021	Randomzied controlled trial, USA	received periodic newsletters	6 m / 23 f<br>6 m / 10 f	Mean: 21.4 ± 13.5<br>Mean: 19.8 ± 13.7	Range: EDSS 5.5-7.5<br>Range: EDSS 5.5-7.5	Mean: 57.8 ± 11.9<br>Mean: 56.0 ± 12.8

Nintendo Wii	Prosperini 2012	Randomized crossover pilot study, Italy	wait-list control	Group A: 5 m / 13 f<br>Group B: 6 m / 12 f	Mean: 12.2 ± 6.0<br>Mean: 9.3 ± 5.3	Median: EDSS 3.0<br>Median: EDSS 3.5	Mean:35.3 ± 8.6<br>Mean: 37.1 ± 8.8
Nintendo Wii	Thomas 2017	Mixed methods pilot study used a single-centre wait-list randomised controlled design, UK	wait-list control	1 m / 14 f<br>2 m / 13 f	Number (5): <1 year: 1(7%), 1-5 years 7 (47%), 6-10 3(20), 11-15 2(13), >16 2(13)<br>Number (5): <1 year 2(13), 1-5 years: 4(27), 6-10: 1(7), 11-15 1(7%), >16: 4(27)	Unknown	Mean: 50.9 ± 8.08<br>Mean: 47.6 ± 9.26
Nintendo Wii	Prosperini 2014	Prospective study, Italy	crossover AND healthy subjects	12 m / 18 f<br>6 m / 9 f	Mean:10.5 ± 5.2	Median EDSS 3.0	Mean: 35.6 ± 7.7.<br>Mean: 34.8 ± 5.6
Preloaded tablet	Kim 2022	Semi-structured interview guide, USA	no control but training at the clinic	TeleCAM 13<br>DirectCAM 7, TeleCAM 1 m / 12 f<br>DirectCAM 0 m / 7 f	Unknown	Median: PDSS Telecam: 1, directCAM: 2	Mean: Telecam 51, Directcam: 47
Home-care activity desk (H-CAD)	Hermens 2008	Randomzied controlled trial, Italy Spain & Belgium	usual care	55 (24 MS)<br>26 (11 MS)	Unknown	Unknown	Mean: 48.1 ± 11.6<br>Mean: 51.9 ± 14.5
Nintendo Wii	Plow 2014	Qualitative study, USA	N/A	7 m / 23 f	Mean: 9.0 ± 6.8	Number(%): PDSS Normal 8 (26.7)	Mean: 43.2 ± 9.3

Mild disability 7 (23.3)<br>Moderate disability 5 (16.7)<br>Gait disability 4 (13.3)<br>Early cane 5 (16.7)<br>Missing 1 (0.03)

Nintendo Wii	Plow 2011	Longitudinal study, USA	N/A	7 m / 23 f	Mean: 9.0 ± 6.8	Number (%): PDSS<br>Normal 8 (26.7)<br>Mild disability 7 (23.3)<br>Moderate disability 5 (16.7)<br>Gait disability 4 (13.3)<br>Early cane 5 (16.7)<br>Missing 1 (0.03)	Mean: 43.2 ± 9.3
